# NONO modulates tumorigenesis and metastatic potential of lung squamous cell carcinoma

**DOI:** 10.3389/fonc.2026.1574103

**Published:** 2026-09-23

**Authors:** Jinlong Huang, Liping Zhu, Weiqing Zhang, Dongliang Lin, Luoyu Lian, Wenshan Hong, Zhendong Xu, Zhoujie Ye

**Affiliations:** 1 Department of Thoracic Surgery, Quanzhou First Hospital Affiliated to Fujian Medical University, Quanzhou, China; 2 Medical Research Center, Fujian Maternity and Child Health Hospital, College of Clinical Medicine for Obstetrics & Gynecology and Pediatrics, Fujian Medical University, Fuzhou, Fujian, China

**Keywords:** apoptosis, cancer, cell proliferation, LUSC, NONO

## Abstract

Lung squamous cell carcinoma (LUSC) is associated with limited therapeutic options and poor clinical prognosis. The Octamer nucleotide--binding transcription factor (NONO), which lacks the POU domain, is implicated in nearly every aspect of gene regulation. It participates in various biological processes including cell proliferation, apoptosis, migration, and DNA damage repair. In this study, we report elevated expression of the NONO gene in LUSC as identified through tumor databases. Our biological function experiments confirmed that NONO promotes the proliferation of LUSC cells by inhibiting apoptosis and enhances the tumor cells’ capabilities for invasion and metastasis. Through the mediation of the PI3K/AKT signaling pathway, NONO regulates downstream molecules such as FOXO1 and GSK3β, which are related to cell proliferation and apoptosis. It participates in the regulation of LUSC cell proliferation, invasion and metastasis, and can serve as a potential clinical target for LUSC, providing ideas for exploring new treatment methods.

## Introduction

According to the latest Global Cancer Statistics report, lung cancer remains the leading cause of cancer-related deaths worldwide, accounting for approximately 1.8 million fatalities annually (1). Non-small cell lung carcinoma (NSCLC) comprises 80% to 85% of all lung cancer cases. Among NSCLC subtypes, adenocarcinoma is the most prevalent (40%), followed by squamous cell carcinoma (25%) (2). Targeted therapies, particularly effective in NSCLC patients harboring specific genetic mutations like those in the EGFR or ALK genes, have shown significant survival benefits, especially in lung adenocarcinoma (LUAD) (3). However, the efficacy of immunotherapies varies widely among patients, with a generally low success rate (4). Compared to LUAD, lung squamous cell carcinoma (LUSC) responds less effectively to NSCLC-targeted treatments, and lacks effective molecular targets for such therapies (5). Consequently, understanding LUSC’s pathogenesis and identifying novel biomarkers and molecular targets for early diagnosis and treatment is imperative.

NONO (non-POU domain containing octamer-binding protein), part of the multifunctional Drosophila behavior/human splicing (DBHS) protein family, is a versatile nuclear protein involved in various gene regulatory functions including RNA splicing, stabilization, and transcriptional regulation (6). Emerging evidence supports NONO’s role in tumorigenesis through its influence on cell proliferation, apoptosis, migration, and DNA damage repair (7–9). Yet, its specific expression and role in LUSC have not been fully elucidated.

This study confirms overexpression of NONO in LUSC tissues, positioning it as a potential oncogene that contributes to LUSC progression. We demonstrate that NONO not only facilitates cell cycle entry and enhances cellular invasion and metastasis but also modulates apoptosis by downregulating Bax and upregulating Bcl-2 proteins through the PI3K/AKT pathway. Collectively, these findings underscore NONO’s pivotal role in LUSC development and suggest its potential as a therapeutic target in LUSC treatment strategies.

## Materials and method

### Acquisition and analysis of gene expression data from public databases

Formalin-fixed paraffin-embedded tissues of lung squamous cell carcinoma and normal lung specimens were retrieved from the Human Protein Atlas database. Immunohistochemical staining for NONO protein was performed following standard procedures, including antigen retrieval, antibody incubation, and DAB visualization. Staining intensity and positive cell proportion were evaluated by blinded pathologists according to HPA criteria. Differences in protein expression between groups were analyzed using Mann–Whitney U test, with a two-sided P<0.05 considered statistically significant.

Transcript-level expression data for the lung cancer cohort were retrieved from The Cancer Genome Atlas (TCGA) database, encompassing 52 normal tissue samples and 503 tumor tissue samples. Tumor specimens were stratified into subgroups based on molecular subtypes (Normal: n=52; NOS: n=480; Basaloid: n=15; Papillary:5; Small cell:n=1) and clinical stages (Normal: n=52; Stage 1: n=243; Stage 2: n=157; Stage 3: n=85; Stage 4: n=7).

Protein expression profiles of the lung cancer cohort were acquired from the Clinical Proteomic Tumor Analysis Consortium (CPTAC) database, including 108 normal tissue samples and 108 tumor tissue samples. These tumor samples were further categorized by clinical stages (Normal: n=108; Stage 1: n=76; Stage 2: n=6; Stage 3: n=6; Stage 4: n=6).

Transcript abundance from TCGA (expressed as transcripts per million) and protein expression from CPTAC (normalized as Z-scores) were visualized using box plots. Statistical comparisons were performed using Student’s t-test for two-group analyses and one-way ANOVA for multiple-group comparisons. Where applicable, P values were adjusted for multiple testing using the Benjamini–Hochberg method. Statistical significance thresholds are indicated in the corresponding figure legends.

### Cell culture and construction

The HEK-293T(#iCell-h237) cell line and the lung cancer cell line SK-MES-1 (#iCell-h192), NCI-H520(#iCell-h238) were obtained from Cellverse CO.,Ltd. HEK-293T and NCI-H520 cells were cultured in high-glucose DMEM medium containing 10% FBS (Gibco, #6123139). SK-MES-1 cells were cultured in MEM medium containing 10% FBS (Gibco, #11095080) in a 5% CO_2_ incubator at 37°C.

For the cell line construction, NONO knockdown was achieved using a lentivirus (Shanghai Gima Pharmaceutical Technology Co., LTD.). Post-infection with lentivirus for 24 hours and subsequent selection with puromycin for a week, the SK-MES-1*^shNONO^* and NCI-H520*^shNONO^* cell lines were established. To generate stable overexpressed cell lines, DNA constructs were transfected into HEK-293T cells using DNA transfection reagent (TengyiBio, #TF20120), along with a lentiviral packaging plasmid. The supernatant containing the virus was collected 48 hours post-transfection. LUSC cells were infected with lentivirus overexpressing NONO to establish a NONO-overexpressing cell line. PI3K inhibitor LY294002 (Cat. No. HY-10108) and PI3K agonist UCL-TRO-1938 (Cat. No. HY-154848) were purchased from MCE. Stock solutions at 50 mM were prepared in DMSO, and the working concentration used in this study was 10 μM.

### RNA extraction, RT-PCR, and RT-qPCR

Total RNA was extracted using a rapid cell total RNA extraction kit (Vazyme, #7E781J3), and cDNA was synthesized using Prime™ RT Master Mix (Takara, #RR036A). Routine RT-PCR was performed using a 2×Hieff^®^ Robust PCR master mixture (Yeasen, #10106ES08). PCR products underwent DNA gel electrophoresis and were visualized with YeaRed nucleic acid gel staining (Yeasen, #10202ES76). RT-qPCR was conducted using SYBR mix (Vazyme, #Q711-03), with results analyzed by the 2^-ΔΔCT^ method. Primer sequences are detailed in **Supplementary Tables S3** and **S4**.

### Immunoblotting

Proteins were extracted using RIPA buffer with protease/phosphatase inhibitors and quantified using a BCA protein quantification kit (Thermo Scientific, #23225). Proteins were subjected to SDS-PAGE, transferred to a PVDF membrane, and blocked with 5% skim milk (BBI Life Science, #F109BA0020). Following incubation with primary and secondary antibodies, bands were visualized using an enhanced chemiluminescence (ECL) kit (Advansta, #180728-03). Primary antibodies were purchased from Cell Signaling Technology, Inc., included NONO(#90336), Bcl2(#15071), Bax(#5023), PARP(#953), Cleaved PARP(#5625), GAPDH(#2118), AKT(#9272), p-AKT(#9271), PI3K(#4292), p-PI3K(#4228), p-FOXO1(#9461), FOXO1(#2880), PUMA(#4976), CDKN1A(#2947), CDKN1B(#3689), Histone H3(#4499) and p-GSK3β(#9336). The quantification method for Western blot bands is described as follows: band gray values were quantified using Image J software, and the relative expression level of each target protein was normalized to the gray value of the corresponding internal reference protein (GAPDH or Histone H3, depending on experimental conditions).

### Cell proliferation and colony formation experiments

For cell proliferation, cells (5×10^3^) were seeded in 96-well plates. Cell viability was assessed using the CCK-8 kit (Dojindo, #CK04) according to the manufacturer’s protocol. For colony formation, cells (1×10^3^) were plated in 6-well plates, cultured for two weeks, fixed with methanol, and stained with 0.1% crystal violet (Solarbo, #C8470).

### Transwell migration and invasion assays

Cells suspended in 200 μL of 5% serum medium were seeded into a Transwell chamber (Corning Incorporated, #08722022) for migration assays or into a chamber precoated with Matrigel for invasion assays. The lower chamber was filled with 600 μL of 20% serum medium. After 24 hours of incubation, cells were fixed with methanol and stained with crystal violet. Positive cells were imaged under a microscope in five independent fields and quantified using ImageJ software.

### Flow cytometry analysis of cell cycle and apoptosis

Cells in logarithmic growth phase were harvested, and 5×10^5^ cells were seeded into each well of a 6-well plate. Cells were collected when reaching 80% confluence. The cells were fixed with 75% ethanol for 4 hours, and the fixative was removed by centrifugation. Subsequently, 50 μL RNase was added and incubated at 37 °C for 30 min, followed by the addition of 200 μL PI solution(Elabscience, China, #AK10096) and incubation in the dark for 30 min. Cell cycle distribution was analyzed using flow cytometry(BD, LSRFortessa). For apoptosis analysis, cells (1×10^6^) were washed with chilled PBS, resuspended in 100 µL of 1% BSA in PBS, and stained with FITC-Annexin-V/PI (Elabscience, China, #AK10150). Cells were incubated at room temperature for 15 minutes in the dark before flow cytometry analysis. Early and late apoptosis were assessed.

### Wound healing assay

Three equidistant lines were marked at the bottom of each well in a 12-well plate. Cells in logarithmic growth phase were seeded appropriately in each well. A horizontal scratch was made using a pipette tip, and the cells were washed with phosphate-buffered saline to remove detached cells. Serum-free medium was then added, and the cells were incubated at 37°C in a 5% CO_2_ environment for measurements at 0 and 72 hours.

### Xenograft tumor assay

All animal experiments were approved by the Ethics Committee of Fujian Maternity and Child Health Hospital, College of Clinical Medicine for Obstetrics, Gynecology and Pediatrics, Fujian Medical University (Approval No.: AEC‑SFY‑XM2025‑267). Six-week-old female BALB/c-nu mice were used for *in vivo* experiments and randomly allocated into multiple groups, with 5 mice in each group. To assess tumorigenicity, LUSC cells (NCI-H520 NC or NCI-H520 shNONO, 5 × 10^6^ cells) were subcutaneously injected into the axilla of the right forelimb of nude mice. Tumor volume was measured every three days using the formula: Volume = (length × width^2^)/2. After two weeks, mice were euthanized and tumors were excised and weighed.

### Hematoxylin and eosin staining

Fresh tumor tissues were fixed in tissue fixative for 48 h. Samples were dehydrated using an automated tissue processor (Leica, Germany), embedded in paraffin, and sectioned. Sections were deparaffinized in xylene, rehydrated through a descending ethanol gradient, and stained with hematoxylin and eosin. After staining, the slides were dehydrated, cleared in xylene, mounted with neutral resin, and visualized under a bright field microscope (Leica, Germany).

### Immunohistochemistry

Paraffin-embedded tissue sections were cut into 4 μm thick slices. Sections were deparaffinized in xylene and rehydrated through a graded ethanol series. Antigen retrieval was performed using citric acid buffer. After blocking with 10% blocking buffer for 20 min, sections were incubated with primary antibodies, followed by HRP-conjugated secondary antibodies. Detection was performed using DAB substrate solution, and nuclei were counter stained with hematoxylin. Finally, sections were dehydrated, mounted with neutral resin, and imaged under a bright field microscope (Leica, Germany). Primary antibodies included mouse anti-human Ki67 (Cat#2009160672j, MXB, China) and NONO (Cat#A5282, ABclonal, China).

### Statistical analysis

Data were analyzed using GraphPad Prism 9 software(GraphPad Software, USA). The data were expressed as the means ± standard deviation (SD) and were obtained from at least three independent experiments. Statistical differences between groups were determined using Two-way ANOVA, with a significance level of *P* < 0.05(notes: *P < 0.05; **P < 0.01; ***P < 0.001;****P < 0.0001, ns, not significant).

## Results

### NONO is overexpressed in LUSC

The complexity of LUSC genomics, combined with limited insights into carcinogenic pathway interactions and the absence of representative animal models, complicates in-depth LUSC research. Aberrant gene expression in tumor cells drives critical oncogenic processes such as proliferation, apoptosis, invasion, and metastasis (10–14). NONO, belonging to the DBHS family of RNA and DNA-binding proteins, is implicated in various gene regulation steps and has been identified as a regulatory factor in numerous tumors.

In this study, we examined NONO expression in both tumor and adjacent normal tissues from LUSC patients using data from the Human Protein Atlas. Immunohistochemical analysis revealed elevated NONO protein levels in tumor tissues compared to adjacent non-tumor tissues (**Figure 1A**). Further analysis of LUSC samples from the TCGA database showed that NONO mRNA levels were significantly higher in tumor tissues than in normal tissues (**Figure 1B**), with a progressive increase across different cancer stages of LUSC (**Figure 1C**). Similar trends were observed across various molecular subtypes of LUSC, where NONO mRNA levels were consistently elevated compared to normal tissue (**Figure 1D**). Correspondingly, protein expression data from the CPTAC dataset confirmed that NONO levels were significantly higher in LUSC tissues (**Figures 1E, F**). Further analysis of LUSC samples in the TCGA database revealed that the level of NONO mRNA was significantly higher in tumors with a history of smoking and those with lymph node metastasis compared to normal tissues (**Figures 1G, H**).

**Figure 1:**
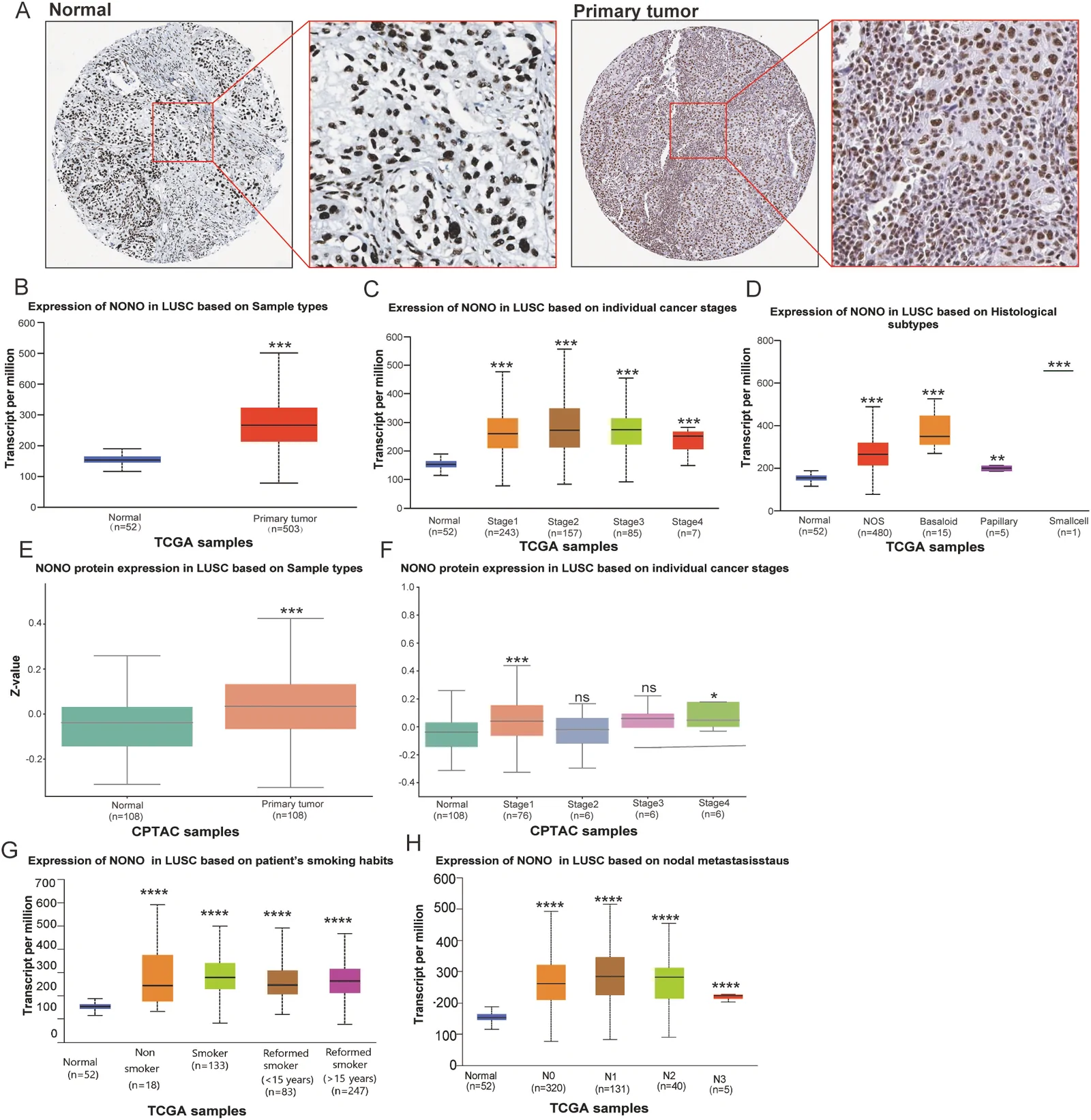
NONO is overexpressed in LUSC tissues and is associated with poor disease prognosis. **(A)** The human protein atlas database analyzed the expression of NONO protein in LUSC. **(B)** Analysis of NONO mRNA levels using TCGA RNA-Seq dataset. **(C)** Expression of NONO in LUSC based on individual cancer stages using TCGA RNA-Seq dataset. **(D)** Expression of NONO in LUSC based on histological. subtypes using TCGA RNA-Seq dataset. **(E, F)** Analysis of NONO mRNA expression levels in LUSC using CPTAC dataset. **(G)** Expression of NONO in LUSC based on patient’s smoking habits using TCGA RNA-Seq dataset. **(H)** Expression of NONO in LUSC based on nodal metastasisstaus using TCGA RNA-Seq dataset. Statistical differences between groups were determined using two-way ANOVA, with a significance level of *P* < 0.05(notes: *P < 0.05; **P < 0.01; ***P < 0.001;****P < 0.0001, ns, not significant.

Collectively, these research results collectively indicate that NONO is significantly upregulated in LUSC tissues, and the overexpression of NONO is closely related to the malignant clinical features and pathological characteristics of LUSC.

### *In vitro* suppression of LUSC cell invasion, metastasis, and proliferation by knockdown of NONO expression

To elucidate the biological functions of NONO in LUSC, we initially knocked down NONO mRNA expressionwoba in the LUSC cell line SK-MES-1 and NCI-H520. Subsequently, we will collectively refer to SK-MES-1 and NCI-H520 as LUSC cells. Western blotting and qPCR analyses confirmed that NONO expression levels were effectively reduced at two distinct knockdown sites (**Figures 2A, B**). Knocking down NONO in LUSC cells inhibited the cell proliferation ability and colony formation ability (**Figure 2C–E**). In the experiment, to prevent the off-target effect, we employed two knockout sites (shNONO#3: GCTGCTACAATGGAAGGAATT and shNONO#4: GCAGGCGAAGTCTTCATTCAT) effectively reduced the expression of NONO in LUSC cells. In LUSC cells, we mainly selected the shNONO#3 site for the subsequent biological function verification.

**Figure 2:**
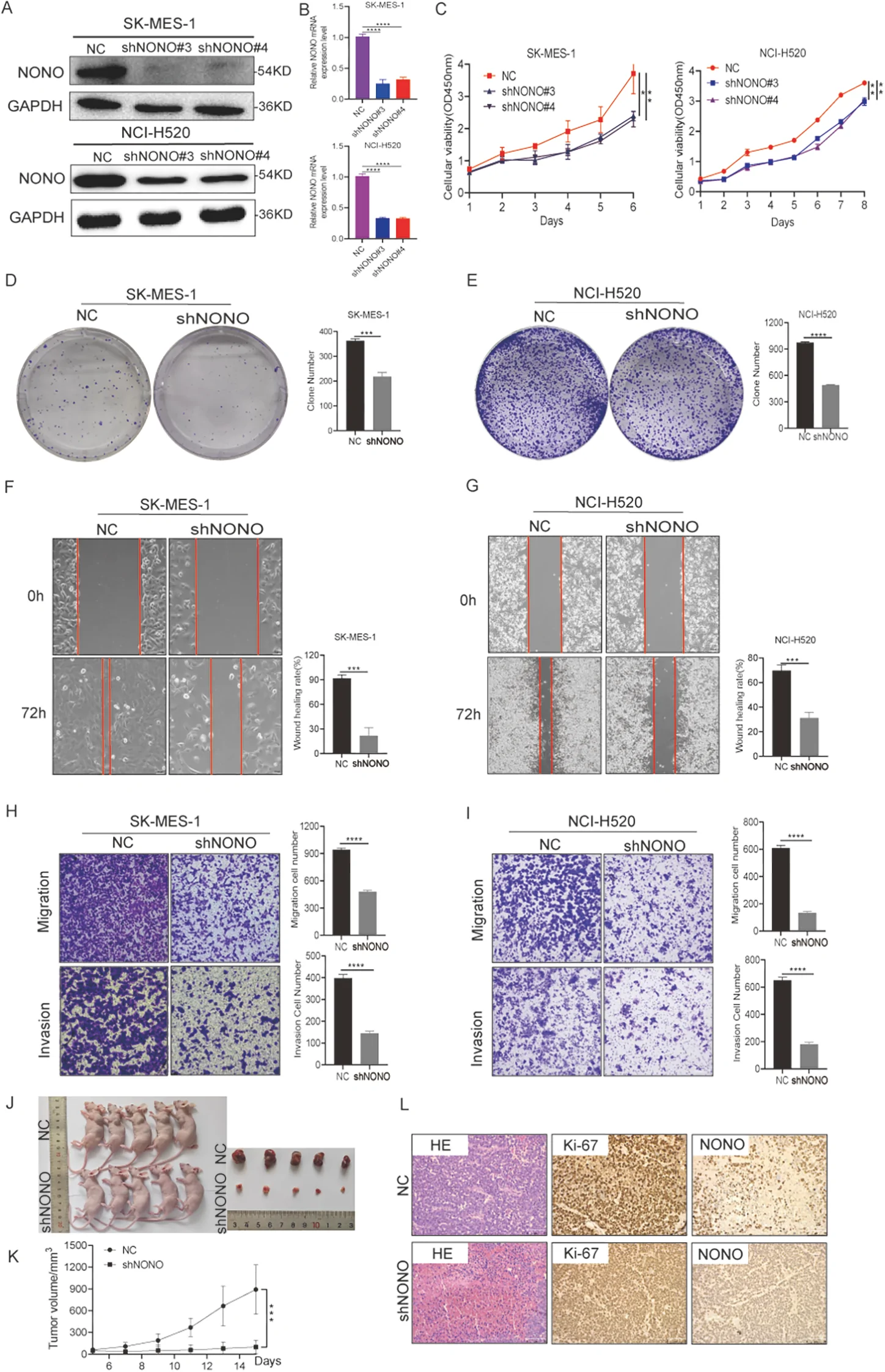
NONO-depleted inhibited the proliferation of LUSC cells. **(A, B)** RT-qPCR and immunoblotting analyses of NONO expression levels in SK-MES-1 and NCI-H520 stably expressing shNC or shNONO (#3and #4). **(C–E)** CCK-8 **(C)** and colony formation **(D, E)** assays were performed using NONO-depleted SK-MES-1 and NCI-H520 cells. **(F, G)** The healing ability of SK-MES-1 and NCI-H520 cells stably expressing shNC or shNONO was determined by cell scratch assay. Scale bar, 100 μm; magnification, ×200. **(H, I)** Transwell migration and invasion assays were conducted with NONO-depleted LUSC cells. Scale bar, 100 μm; magnification, ×200. **(J)** NCI-H520 cells NC or shNONO#3 were seeded into the mammary fat pad of BALB/c-nu mice (n = 5). Representative images of tumors *in situ* and excised tumors are shown. **(K)** The tumor growth curve, drawn according to the average percentage. Change of the tumor volume. **(L)** H&E staining and immunohistochemical detection of Ki-67 and NONO in tumor tissues, Scale bar, 75 μm; magnification, ×200. (Statistical differences between groups were determined using two-way ANOVA, with a significance level of *P* < 0.05(notes: *P < 0.05; **P < 0.01; ***P < 0.001;****P < 0.0001, ns, not significant.) Data are presented as mean ± SD (n = 3).

Given the high invasiveness and substantial risk of distant metastasis characteristic of LUSC, we further investigated the impact of NONO down-regulation on the migration and invasion abilities of LUSC cells. The wound healing assay revealed a decreased scratch recovery capability in LUSC cells post-NONO downregulation, suggesting a diminished cell migration capacity (**Figures 2F, G**). Consistently, Transwell migration and invasion assays corroborated these findings, showing a significant reduction in the invasive and metastatic potentials of LUSC cells following NONO knockdown (**Figures 2H, I**). To assess the *in vivo* relevance, we implanted NONO-deficient and control cells subcutaneously into BALB/c-nu mice. Consistent with the *in vitro* data, tumors derived from NONO knock­down cells grew significantly slower than controls (**Figures 2J, K**). The immunohistochemical experiment results showed that in the tumor tissues with reduced NONO expression, the expression of Ki67 was significantly lower than that in the control group (**Figure 2L**).

These findings collectively propose that reduced expression of NONO, a potential oncogene in LUSC, can inhibit not only cell proliferation but also the invasive and metastatic behaviors of tumor cells.

### Inhibition of NONO expression induces cell cycle arrest and promotes apoptosis in LUSC cells

Tumor cell cycles often proceed at an accelerated and uncontrolled rate, facilitating limitless cell proliferation. In our analysis, cell cycle phases were distinctly altered by NONO knockdown. Compared to the empty vector control, a reduction in the G0/G1 phase and G2/M phase population and an increase in the S phase population were observed (**Figure 3A**), indicating a blockade during the DNA synthesis phase. This disruption slows down cell proliferation efficiency. Further investigations revealed an enhancement in apoptosis rates following NONO knockdown, particularly in late-stage apoptosis (**Figure 3B**), illustrating that NONO suppression leads to both cell cycle arrest and increased apoptosis in LUSC cells.

**Figure 3:**
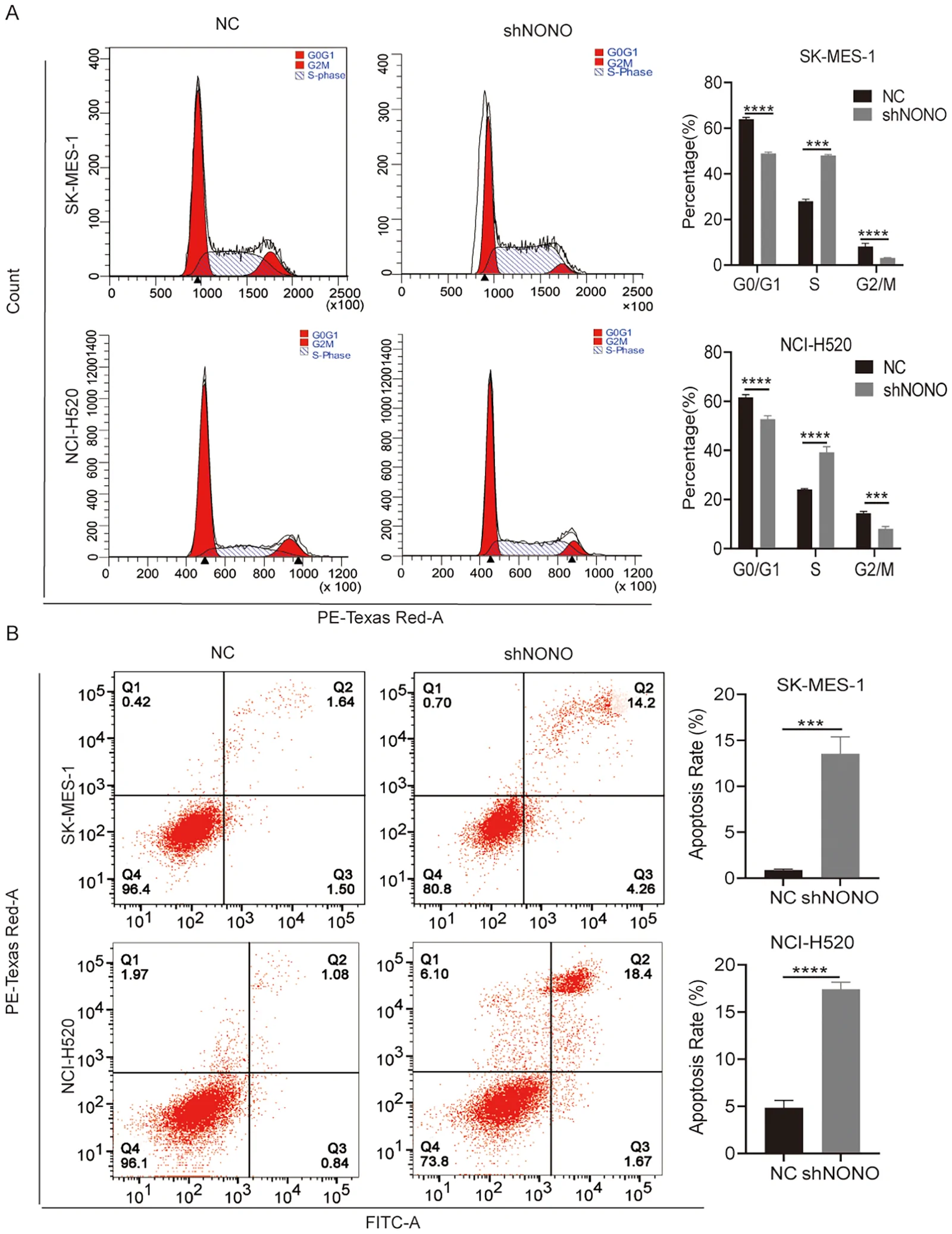
NONO-depleted leads to depleted cell cycle and depleted cell apoptosis. **(A)** Flow cytometry was used to detect cell cycle with NONO-depleted SK-MS-1 and NCI-H520 cells. **(B)** Flow cytometry was used to detect apoptosis with NONO-depleted SK-MES-1 and NCI-H520 cells. (Statistical differences between groups were determined using Two-way ANOVA, with a significance level of *P* < 0.05(notes: *P < 0.05; **P < 0.01; ***P < 0.001;****P < 0.0001, ns, not significant.) Data are presented as mean ± SD (n = 3).

### Overexpression of NONO enhances invasion, metastasis, and proliferation in LUSC cells

To ascertain the full biological impact of NONO on LUSC cells, we conducted experiments to overexpress the NONO gene. Using a pLVX-puro vector for overexpression, we observed marked increases in both protein and mRNA levels of NONO compared to controls (**Figures 4A, B**). This upregulation substantially enhanced cellular proliferation (**Figures 4C, D**) and colony formation abilities (**Figures 4E, F**). Wound healing assays demonstrated improved migration capabilities post-NONO overexpression (**Figures 4G, H**). Similarly, Transwell migration and invasion assays corroborated these findings, showing significant enhancements in the invasive and metastatic potential of LUSC cells upon NONO overexpression (**Figures 4I, J**). These effects were consistent in different LUSC cells with NONO knockdown, confirming the key role of NONO in regulating the proliferation, invasion and metastasis behaviors of LUSC cells.

**Figure 4:**
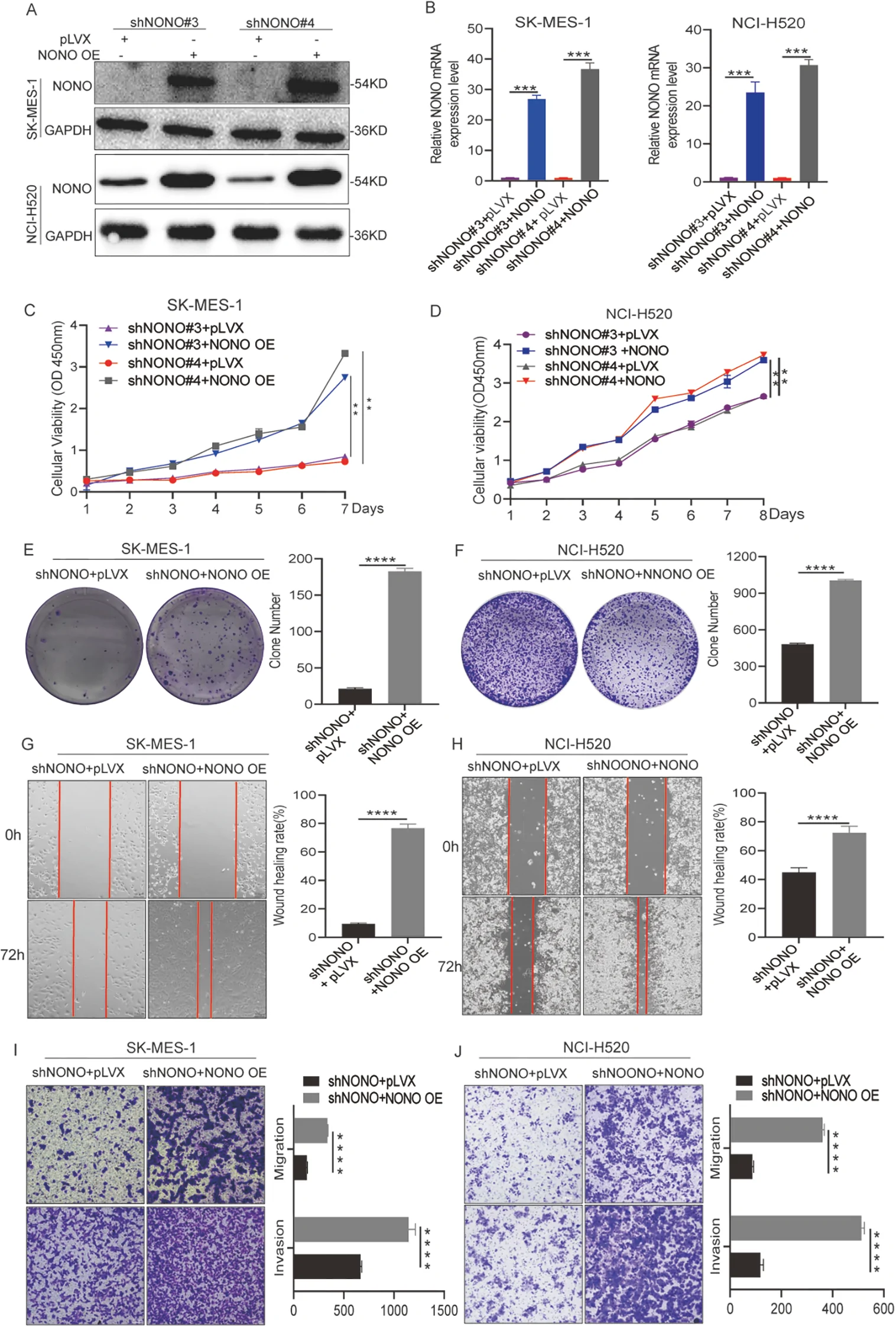
NONO overexpression promotes the proliferation of LUSC cells. **(A, B)** RT-qPCR(B) and immunoblotting(A) analyses of NONO expression levels in LUSC cells stably expressing pLVX or NONO. **(C, D)** CCK-8 assays were performed using NONO-overexpression SK-MES-1 or NCI-H520 cells. **(E, F)** Colony formation assays were performed using NONO-overexpression LUSC cells. **(G, H)** The healing ability of LUSC cells stably expressing pLVX or NONO was determined by cell scratch assay. Scale bar, 100 μm; magnification, ×200. **(I, J)** Transwell migration and invasion assays were conducted with NONO-overexpression LUSC cells. Scale bar, 100 μm; magnification, ×200. (Statistical differences between groups were determined using two-way ANOVA, with a significance level of *P* < 0.05(notes: *P < 0.05; **P < 0.01; ***P < 0.001;****P < 0.0001, ns, not significant.) Data are presented as mean ± SD (n = 3).

### Impact of NONO expression on cell proliferation and apoptosis

To maintain organismal homeostasis and curb unchecked cellular proliferation, a dynamic balance between cell proliferation and apoptosis is essential. Prior research has demonstrated that downregulation of NONO in LUSC cells leads to increased apoptosis and reduced proliferation rates. Subsequent studies involving the expression of exogenous NONO showed that, through flow cytometry assessment, the population of S phase and G2/M phase cells increased. This indicates that the cell division rate has increased compared to the control group carrying an empty vector (**Figures 5A, B**).This suggests an enhanced cell proliferation ability. Apoptosis assays showed that NONO overexpression significantly reduced apoptosis rates, particularly during the mid and late stages, compared to the control group (**Figures 5C, D**).

**Figure 5:**
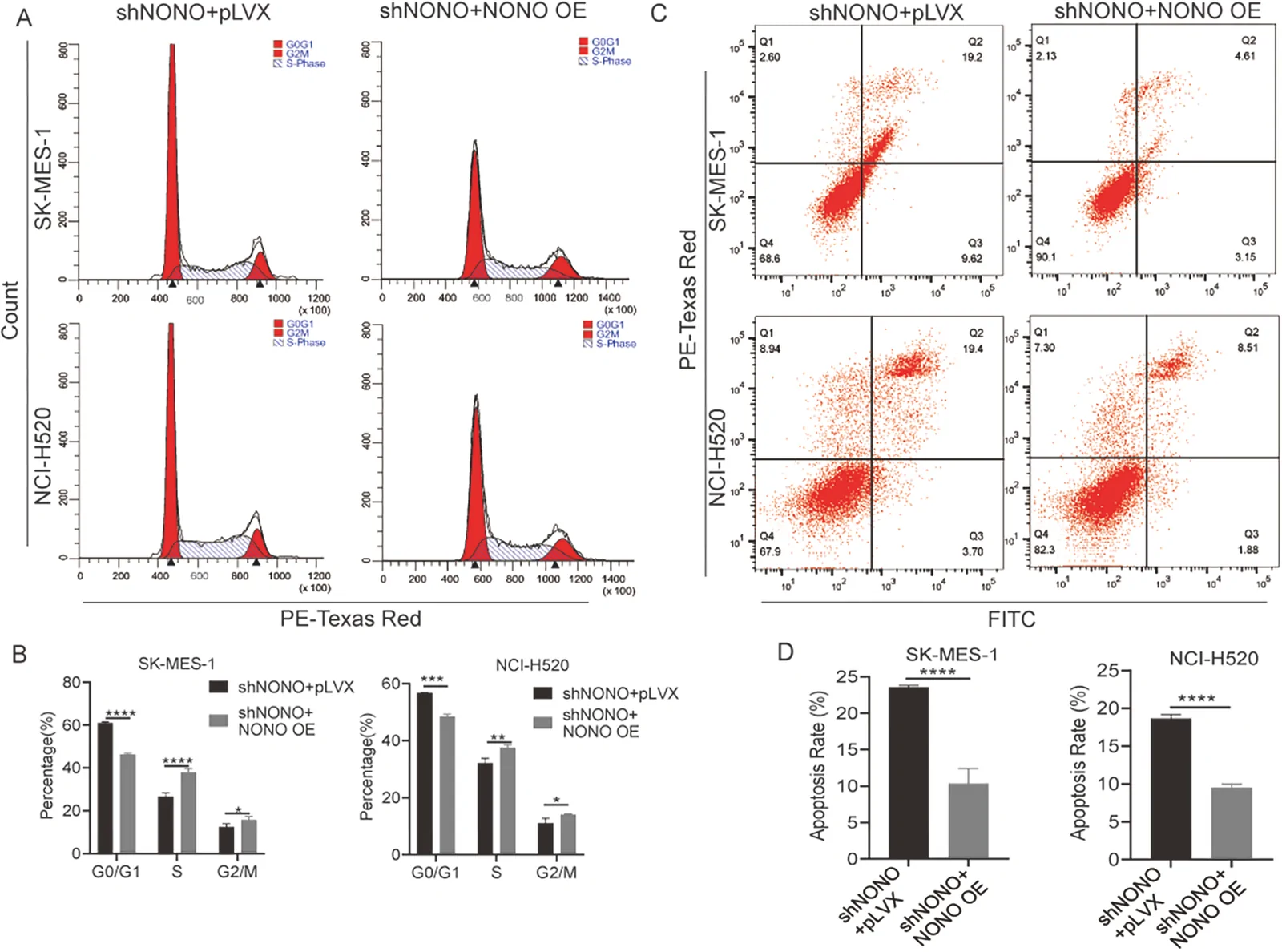
Overexpression of NONO inhibited apoptosis of SK-MES-1 cells. **(A)** Flow cytometry was used to detect apoptosis with NONO-overexpression SK-MES-1 cells. **(B)** Cell-cycle distribution statistics showing the percentage of cells in each cell-cycle phase. **(C)** Flow cytometry was used to detect cell cycle with NONO-overexpression SK-MS-1 cells. **(D)** Quantitative statistics of apoptotic cell proportions. (Statistical differences between groups were determined using Two-way ANOVA, with a significance level of *P* < 0.05(notes: *P < 0.05; **P < 0.01; ***P < 0.001;****P < 0.0001, ns, not significant.) Data are presented as mean ± SD (n = 3).

These findings underscore the critical role of NONO expression in modulating both the cell cycle and apoptosis in LUSC cells. Specifically, decreased NONO expression facilitates apoptosis, whereas its overexpression promotes proliferation and suppresses apoptotic processes.

### NONO regulates the protein expression of Bax, Bcl-2 and PARP

Bax and Bcl-2 proteins are members of the Bcl-2 family that regulate mitochondrial membrane permeability and consequently determine the initiation of apoptosis (15). Bax is a pro-apoptotic protein that increases mitochondrial membrane permeability, releasing cytochrome C and other apoptosis-inducing factors into the cytoplasm (16). Conversely, Bcl-2 is an anti-apoptotic protein that protects cells from apoptosis by inhibiting the activity of pro-apoptotic factors like Bax.

Upon NONO knockdown in LUSC cells, Bax expression was significantly up-regulated, while the expression of the apoptosis-inhibiting Bcl-2 protein decreased markedly compared to the control group (**Figures 6A, B**). These changes align with our earlier experimental results. PARP, a substrate of caspase and a crucial player in cell stability and survival, also showed decreased expression upon NONO knockdown, with an increase in cleaved PARP, indicating enhanced apoptotic activity (17). Conversely, exogenous overexpression of the NONO gene resulted in a notable down-regulation of Bax protein levels and an increase in Bcl-2 expression, which inhibits apoptosis initiation. Similarly, the levels of cleaved PARP were significantly reduced (**Figures 6C, D**).

**Figure 6:**
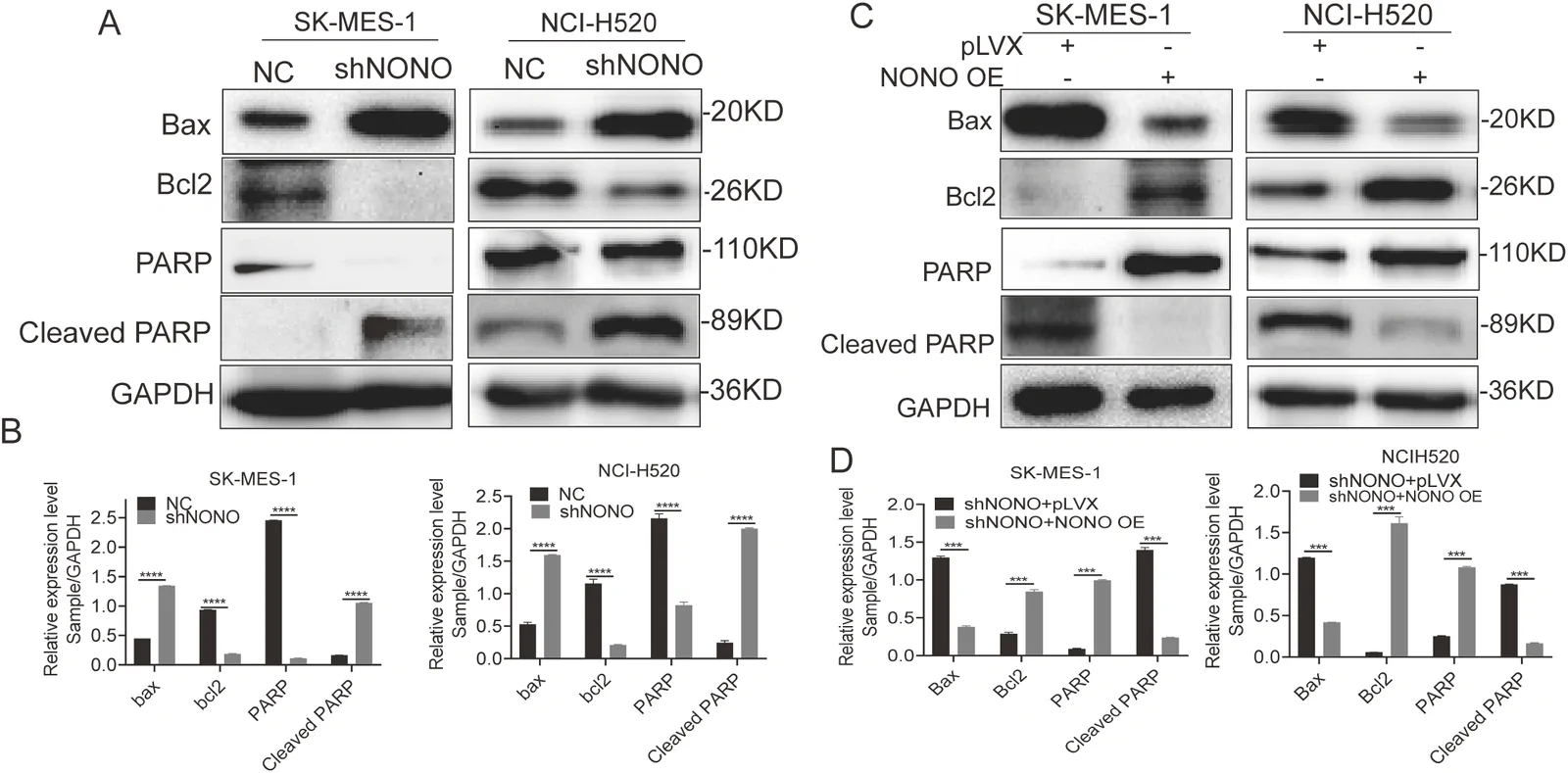
NONO affects the expression of apoptosis-related proteins in LUSC cells. **(A)** In SK-MES-1 and NCI-H520 cells, the absence of NONO can upregulate the expression of Bax and cleaved PARP proteins. **(B)** Statistical analysis of protein expression. **(C)** Exogenous expression of NONO in LUSC cells can inhibit the expression of Bax and cleaved PARP proteins, and this expression promotes the apoptotic process. **(D)** Statistical analysis of protein expression. (Statistical differences between groups were determined using Two-way ANOVA, with a significance level of *P* < 0.05(notes: *P < 0.05; **P < 0.01; ***P < 0.001;****P < 0.0001, ns, not significant.) Data are presented as mean ± SD (n = 3).

### NONO regulates the functions of LUSC cells via the PI3K/AKT signaling pathway

The mechanism by which NONO regulates the expression of apoptosis-related proteins in LUSC cells requires further elucidation. Previous studies have shown that NONO promotes cell proliferation via the PI3K/AKT pathway (18, 19). After knocking down NONO in LUSC cells, we observed a significant downregulation in the expression levels of phosphorylated PI3K and AKT, as well as a marked reduction in the phosphorylation of downstream factors FOXO1 and GSK3β. Meanwhile, nuclear FOXO1 was found to be significantly upregulated upon analysis (**Figures 7A, B**). FOXO1, a key transcription factor in the regulation of apoptosis, can directly initiate and modulate apoptotic processes by controlling the expression of apoptosis-related genes such as PUMA, Bax and Bcl-2 (20, 21). Consistent with these findings, depletion of NONO facilitates nuclear translocation of FOXO1 and upregulates PUMA and CDKN1A protein expression, which consequently promotes cellular apoptosis (**Figures 7A, B**). The PI3K/AKT pathway inhibits the nuclear localization and transcriptional activity of FOXO1 through phosphorylation, thereby negatively regulating FOXO1-mediated apoptosis. GSK3β is also involved in regulating the activity of Bax and Bcl-2 proteins to mediate apoptosis (22, 23). Rescue experiments with NONO in LUSC cells led to the upregulation of phosphorylation levels of PI3K, AKT, FOXO1, and GSK3β. FOXO1 was retained in the cytoplasm, accompanied by decreased expression of the pro-apoptotic proteins PUMA and CDKN1A. (**Figures 7C, D**). We further validated the protein expression levels of PI3K/AKT/FOXO1/GSK3β.

**Figure 7:**
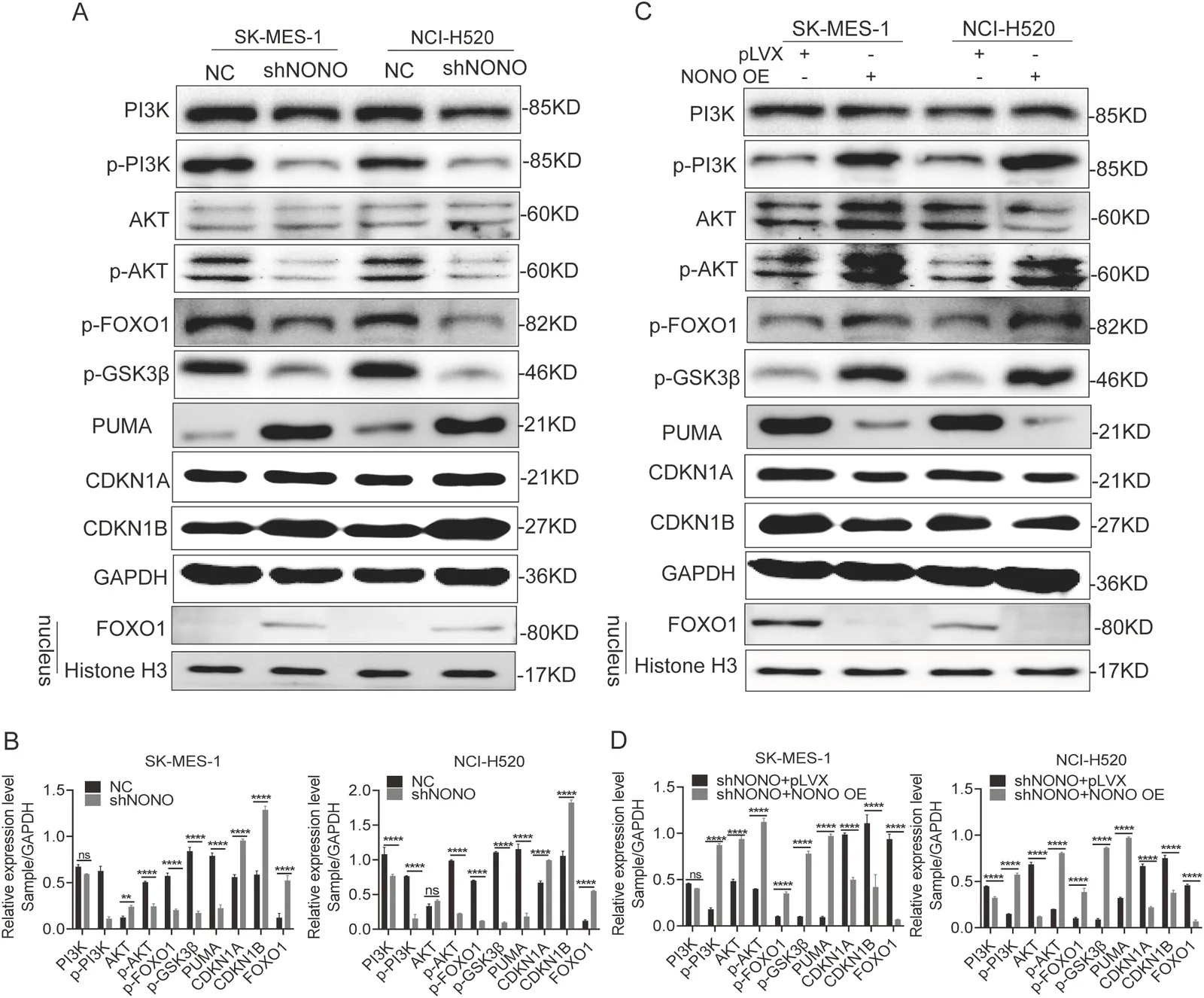
The effect of NONO on the expression of proteins related to the PI3K/AKT signaling pathway in LUSC cells. **(A)** NONO-depleted in LUSC can inhibit the activation of PI3K/AKT. **(B)** Statistical analysis of protein expression. **(C)** Exogenous expression of NONO in LUSC cells can promote the phosphorylation of PI3K/AKT and activate downstream protein expression. **(D)** Statistical analysis of protein expression. (Statistical differences between groups were determined using Two-way ANOVA, with a significance level of *P* < 0.05(notes: *P < 0.05; **P < 0.01; ***P < 0.001;****P < 0.0001, ns, not significant.) Data are presented as mean ± SD (n = 3).

To further verify that NONO regulates cell apoptosis through the PI3K/AKT pathway, we conducted rescue experiments on the cells that were treated with the PI3K inhibitor LY294002 and the PI3K agonist UCL-TRO-1938 respectively. After treating LUSC cells with LY294002, the proliferation (**Figure 8A**) and colony formation ability of NC and shNONO LUSC cells were both inhibited (Figure C). After treating the cells with UCL-TRO-1938, the reduced proliferation and colony formation ability caused by NONO knockdown could be reversed (**Figures 8B, C**). The cell cycle detection results showed that LY294002 could induce S-phase arrest of the cells, while UCL-TRO-1938 could reverse this cycle arrest effect, causing the proportions of S-phase and G2/M-phase cells to increase simultaneously (**Figure 8D**). The cell apoptosis results indicated that UCL-TRO-1938 could reduce the apoptosis level of cells in the NONO knockdown group (**Figure 8E**). After LUSC cells were pretreated with PI3K inhibitors, it was observed that the phosphorylation levels of PI3K, AKT and GSK3β decreased in both the NC group and the NONO knockout group (**Figure 8F**). After pretreatment with PI3K agonists, the phosphorylation levels of PI3K, AKT and GSK3β increased in the NC group, and a similar trend was observed in the NO knockout group, but the total protein levels of PI3K and AKT remained unchanged in both groups (**Figure 8G**). In summary, these data indicate that NONO regulates the activity of PI3K/AKT through post-translational phosphorylation levels, rather than altering their total protein content.

**Figure 8:**
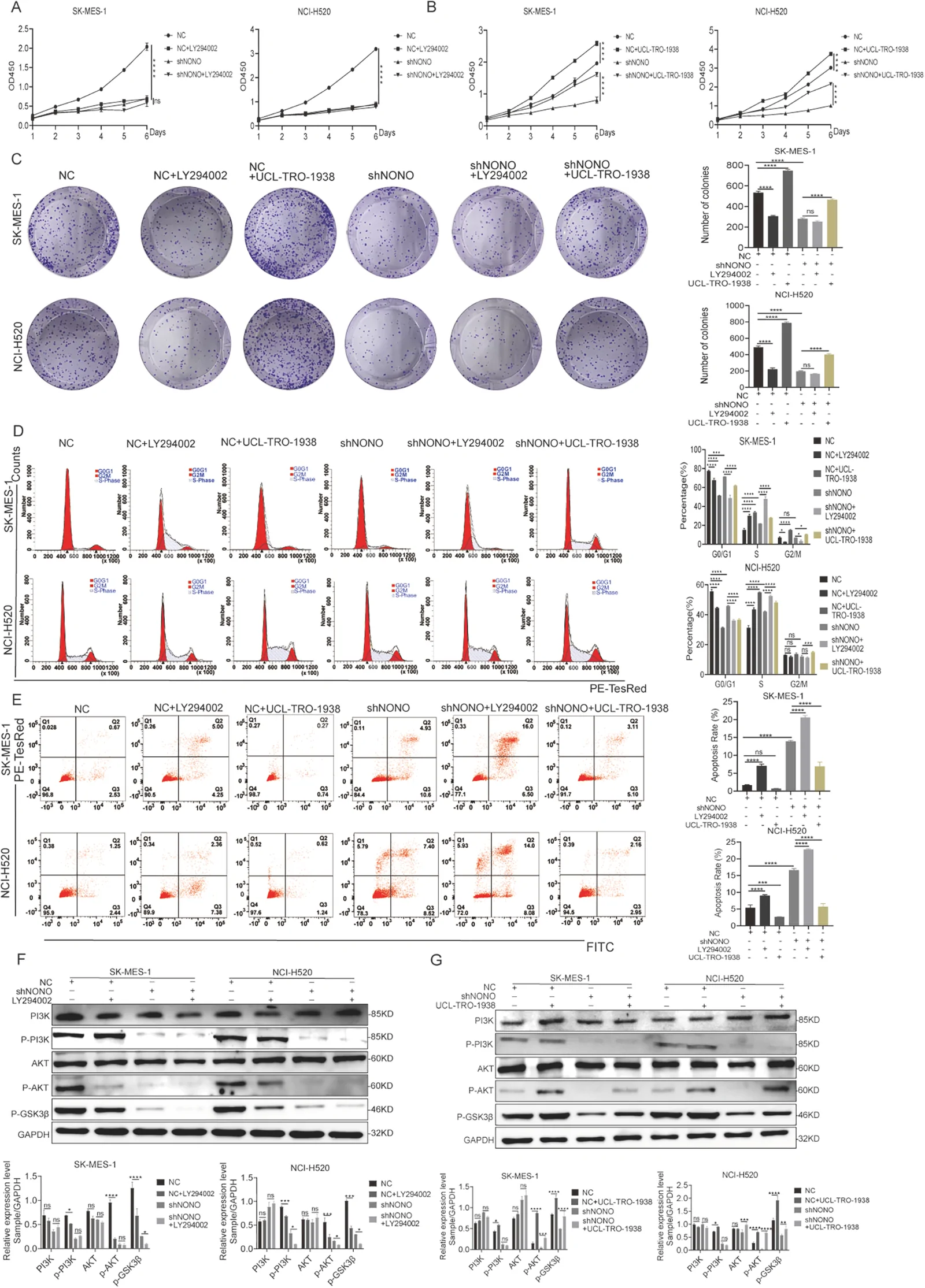
NONO depletion modulates the responsiveness of the PI3K/AKT pathway toward PI3K agonists or inhibitors. **(A)** CCK-8 assay for the effects of PI3K inhibitor on LUSC cell proliferation. **(B)** CCK-8 assay for the effects of PI3K agonist on LUSC cell proliferation. **(C)** Effects of PI3K agonist or inhibitor on colony-forming ability of LUSC cells. **(D)** Regulatory effects of PI3K agonist or inhibitor on cell-cycle progression in LUSC cells. **(E)** Regulatory effects of PI3K inhibitor or agonist on apoptosis in LUSC cells. **(F)** Western blot was used to examine changes in PI3K/AKT signaling axis protein levels in LUSC cells treated with 10 μM PI3K inhibitor LY294002 for 3 hours. And statistical analysis of protein expression. **(G)** The influence of PI3K agonist UCL-TRO-1938 (10 μM, treatment for 20 min) on PI3K/AKT pathway proteins in NONO-silenced LUSC cells. And statistical analysis of protein expression. (Statistical differences between groups were determined using Two-way ANOVA, with a significance level of *P* < 0.05(notes: *P < 0.05; **P < 0.01; ***P < 0.001;****P < 0.0001, ns, not significant.).Data are presented as mean ± SD (n = 3).

In summary, changes in NONO expression significantly affect the expression of apoptosis-related proteins. The underlying mechanism may involve NONO-mediated activation of the PI3K/AKT signaling pathway, which subsequently influences downstream proliferation and apoptosis-related signaling proteins FOXO1 and GSK3β, thereby impacting cell proliferation and survival.

## Discussion

Approximately 80%-85% of lung cancer patients are diagnosed with non-small cell lung cancer (NSCLC), and LUSC accounts for about 20%-30% of these NSCLC cases (24), making it a prevalent pathological subtype of lung cancer (25). In clinical practice, targeted therapy has become a critical treatment modality for LUSC patients, complementing traditional surgery and chemoradiotherapy. Notably, epidermal growth factor receptor tyrosine kinase inhibitors (EGFR-TKIs) have brought significant benefits to lung adenocarcinoma patients, substantially improving their treatment outcomes and survival rates. However, research on LUSC lags far behind that on lung adenocarcinoma, and to date, there are no widely recognized and effective targeted therapies available to guide clinical practice for LUSC (26). This research gap is presumably attributed to an inadequate understanding of the biological characteristics of LUSC. Therefore, exploring the molecular biology of LUSC through genetic studies and identifying differentially expressed genes are crucial approaches, which hold significant potential for discovering new therapeutic targets for this disease. It should be noted that this study only initially analyzed the association between NONO expression and clinical characteristics in LUSC using public databases, without systematically evaluating the efficacy of NONO as a prognostic marker, such as through ROC curves and sensitivity analysis. In the future, expanding the clinical sample size will be necessary to further verify the clinical value of NONO as a prognostic or discriminative marker for LUSC.

NONO is a type of RNA binding protein (RBP), and accumulating evidence has shown that the dysregulation of some RBPs with splicing functions can promote cancer development. Specifically, emerging studies have demonstrated that NONO is overexpressed in various malignant tumors, including bladder cancer (27), lung cancer (28), prostate cancer, and esophageal squamous cell carcinoma (ESCC) (29). In addition, NONO protein levels have been identified as an independent prognostic factor in certain cancers. However, the expression pattern of NONO varies across different cancer types; for instance, it is typically downregulated in estrogen receptor (ER)-negative breast cancer. At the genetic level, renal cell carcinoma (RCC) is closely associated with TFE3 (transcription factor E3)-NONO fusion (30), indicating that NONO may exert diverse pathological effects through different mechanisms in various cancers.

In this study, we identified NONO as a potential prognostic marker for LUSC through a series of analyses and experiments. First, data from tumor databases showed that the mRNA and protein expression levels of the NONO gene in LUSC patients were significantly higher than those in adjacent normal tissues (**Figure 1**), suggesting an abnormal upregulation of NONO in LUSC. Since uncontrolled proliferation and enhanced invasiveness are key features that increase tumor malignancy, we further explored the biological functions of NONO in LUSC cells. The results showed that suppressing NONO expression in LUSC cells significantly impaired cellular biological functions, specifically promoting cell apoptosis while inhibiting cell proliferation and metastasis (**Figure 2**). To further confirm that the observed phenotypic changes were specifically mediated by NONO downregulation, we performed rescue experiments: exogenous overexpression of the NONO gene in LUSC cells reversed the effects caused by NONO downregulation, restoring the cells’ abilities to proliferate, invade, and metastasize (**Figure 4**). Moreover, we explored the potential mechanism by which NONO regulates cell apoptosis: downregulating NONO expression promoted the expression of the pro-apoptotic protein Bax and the cleavage of PARP, thereby initiating the apoptosis process (**Figure 6**); on the contrary, overexpression of NONO upregulated Bcl-2 expression, which in turn inhibited cell apoptosis (**Figure 6**). These results collectively confirm that NONO plays a regulatory role in LUSC cell apoptosis.

Although this study has confirmed that the alteration of NONO expression can significantly affect the activation state of the PI3K/AKT/FOXO1/GSK3β signaling pathway, indicating that this signaling axis may be an important upstream pathway that mediates the cell apoptosis process regulated by NONO in LUSC, there are still some limitations in the current research. Firstly, this study only preliminarily revealed the association between this pathway and the function of NONO, while the specific regulatory details of NONO and the PI3K/AKT/FOXO1/GSK3β pathway and the causal relationship between them have not been fully clarified. Therefore, more in-depth mechanism studies need to be carried out in the future, and multiple experimental techniques should be combined to further verify and clarify the molecular mechanism by which NONO regulates cell apoptosis in LUSC. Secondly, NONO is a widely expressed basal layer RNA-binding protein. How to limit the targeted intervention to tumor cells and not damage normal tissues is a special issue that needs to be particularly focused on for NONO to truly become a therapeutic target for LUSC. We have summarized three clinically feasible strategies to achieve tumor-restricted NONO intervention: nanoparticle nucleic acid delivery technology with antigen modification targeting LUSC surface biomarkers, NONO inhibitor prodrugs triggered by the tumor microenvironment, and selective PROTAC degrader specifically for degrading the modified tumor NONO complex. These strategies have been successfully applied to other widely expressed RNA-binding cancer proteins, such as HuR and hnRNPM (31–34). These research results can provide research directions for clinical application transformation of NONO as a clinical treatment target for LUSC.

Collectively, our research results reveal a previously unrecognized role of NONO in the progression of LUSC. Specifically, elevated NONO expression in LUSC can promote tumor cell proliferation, inhibit cell apoptosis, and enhance the invasiveness and metastatic potential of tumor cells. Based on these findings, NONO may serve as a viable therapeutic target for LUSC, providing a new direction for the development of targeted therapies for this disease.

## Data Availability

The original contributions presented in the study are included in the article/**Supplementary Material**. Further inquiries can be directed to the corresponding authors.

## References

[1] Sung H Ferlay J Siegel RL Laversanne M Soerjomataram I Jemal A . Global cancer statistics 2020: GLOBOCAN estimates of incidence and mortality worldwide for 36 cancers in 185 countries. CA: A Cancer J For Clin. (2021) 71:209–49. doi: 10.3322/caac.21660 33538338

[2] Bajbouj K Al-Ali A Ramakrishnan RK Saber-Ayad M Hamid Q . Histone modification in NSCLC: molecular mechanisms and therapeutic targets. Int J Mol Sci. (2021) 22:11701. doi: 10.3390/ijms222111701 34769131 PMC8584007

[3] Thai AA Solomon BJ Sequist LV Gainor JF Heist RS . Lung cancer. Lancet. (2021) 398:535–54. doi: 10.1016/s0140-6736(21)00312-3 34273294

[4] Yang M Lin C Wang Y Chen K Zhang H Li W . Identification of a cytokine-dominated immunosuppressive class in squamous cell lung carcinoma with implications for immunotherapy resistance. Genome Med. (2022) 14:72. doi: 10.1186/s13073-022-01079-x 35799269 PMC9264601

[5] Reck M Remon J Hellmann MD . First-line immunotherapy for non-small-cell lung cancer. J Clin Oncol Off J Am Soc Clin Oncol. (2022) 40:586–97. doi: 10.1200/JCO.21.01497 34985920

[6] Knott GJ Bond CS Fox AH . The DBHS proteins SFPQ, NONO and PSPC1: a multipurpose molecular scaffold. Nucleic Acids Res. (2016) 44:3989–4004. doi: 10.1093/nar/gkw271 27084935 PMC4872119

[7] Pavao M Huang YH Hafer LJ Moreland RB Traish AM . Immunodetection of nmt55/p54nrb isoforms in human breast cancer. BMC Cancer. (2001) 1:15. doi: 10.1186/1471-2407-1-15 11710964 PMC59838

[8] Bray F Ferlay J Soerjomataram I Siegel RL Torre LA Jemal A . Global cancer statistics 2018: GLOBOCAN estimates of incidence and mortality worldwide for 36 cancers in 185 countries. CA: A Cancer J For Clin. (2018) 68:394–424. doi: 10.3322/caac.21492 30207593

[9] Odashiro M Hans Filho G Pereira PR Castro AR Stief AC Pontes ER . Melanoma inhibitory activity in Brazilian patients with cutaneous melanoma. Anais Brasileiros Dermatol. (2015) 90:327–32. doi: 10.1590/abd1806-4841.20153248 26131861 PMC4516101

[10] Li M Tian H Zhou Z Jiang Y Guo X Qin W . O-GlcNAcylation of NONO mediates alternative splicing of SETMAR and facilitates NHEJ repair. Genome Biol. (2026) 27:26. doi: 10.1186/s13059-026-03930-5 41535889 PMC12888201

[11] Iino K Mitobe Y Ikeda K Takayama KI Suzuki T Kawabata H . RNA-binding protein NONO promotes breast cancer proliferation by post-transcriptional regulation of SKP2 and E2F8. Cancer Sci. (2020) 111:148–59. doi: 10.1111/cas.14240 31733123 PMC6942431

[12] Cheng R Zhu S Guo S Min L Xing J Guo Q . Downregulation of NONO induces apoptosis, suppressing growth and invasion in esophageal squamous cell carcinoma. Oncol Rep. (2018) 39:2575–83. doi: 10.3892/or.2018.6334 29620226 PMC5983930

[13] Kim Y Lee KT Kim HB Jung H Ko JY Kweon TH . O-GlcNAcylation of NONO regulates paraspeckle component assembly and contributes to colon cancer cell proliferation. Cell Death Discov. (2025) 11:234. doi: 10.1038/s41420-025-02405-z 40360465 PMC12075841

[14] Li Q Ci H Zhao P Yang D Zou Y Chen P . NONO interacts with nuclear PKM2 and directs histone H3 phosphorylation to promote triple-negative breast cancer metastasis. J Exp Clin Cancer Res. (2025) 44:90. doi: 10.1186/s13046-025-03343-5 40059196 PMC11892261

[15] Renault TT Dejean LM Manon S . A brewing understanding of the regulation of Bax function by Bcl-xL and Bcl-2. Mech Ageing Dev. (2017) 161:201–10. doi: 10.1016/j.mad.2016.04.007 27112371

[16] Spitz AZ Gavathiotis E . Physiological and pharmacological modulation of BAX. Trends Pharmacol Sci. (2022) 43:206–20. doi: 10.1016/j.tips.2021.11.001 34848097 PMC8840970

[17] Wang H Ge W Jiang W Li D Ju X . SRPK1-siRNA suppresses K562 cell growth and induces apoptosis via the PARP-caspase3 pathway. Mol Med Rep. (2018) 17:2070–6. doi: 10.3892/mmr.2017.8032 29138847

[18] Zhao G Liu R Ge L Qi D Wu Q Lin Z . NONO regulates m5C modification and alternative splicing of PTEN mRNAs to drive gastric cancer progression. J Inflammation (London). (2025) 44:81. doi: 10.1186/s13046-024-03260-z 40033337 PMC11877715

[19] Lei YQ Ye ZJ Wei YL Zhu LP Zhuang XD Wang XR . Nono deficiency impedes the proliferation and adhesion of H9c2 cardiomyocytes through Pi3k/Akt signaling pathway. Sci Rep. (2023) 13:7134. doi: 10.1038/s41598-023-32572-x 37130848 PMC10154399

[20] Zhang Y Wang H Wang Y Ma B . FOXO1 mediates miR-99a-5p/E2F7 to restrain breast cancer cell proliferation and induce apoptosis. BMC Cancer. (2025) 25:747. doi: 10.1186/s12885-025-14111-1 40264026 PMC12013025

[21] Xie W Zhang L Jiao H . Chaperone-mediated autophagy prevents apoptosis by degrading BBC3/PUMA. Autophagy. (2015) 11:1623–35. doi: 10.1080/15548627.2015.1075688 26212789 PMC4590652

[22] Zhang D Shi C Zhang Q Wang Y Guo J Gong Z . Inhibition of GSK3β activity alleviates acute liver failure via suppressing multiple programmed cell death. J Inflammation (London). (2023) 20:24. doi: 10.1186/s12950-023-00350-1 37443080 PMC10347874

[23] Qiu Y Li Y Chai M Hua H Wang R Waxman S . The GSK3β/Mcl-1 axis is regulated by both FLT3-ITD and Axl and determines the apoptosis induction abilities of FLT3-ITD inhibitors. Cell Death Discov. (2023) 9:44. doi: 10.1038/s41420-023-01317-0 36739272 PMC9899255

[24] Zhang NN Wang AM . Progression on molecular pathology of squamous cell lung cancer. Lin Chuang Zhong Liu Xue Za Zhi. (2013) 18:947–50. doi: 10.3969/j.issn.1009-0460.2013.10.017

[25] Ma L Zhang S . Research Progress of Molecular Targeted Therapy in Lung Squamous Cell Carcinoma [J]. Chinese Journal of Lung Cancer, (2013) 2013(12):671–5. doi: 10.3779/j.issn.1009-3419.2013.12.10 24345494 PMC6000638

[26] Shi X Wu H Lu J Duan H Liu X Liang Z . Screening for major driver oncogene alterations in adenosquamous lung carcinoma using PCR coupled with next-generation and Sanger sequencing methods. Sci Rep. (2016) 6:22297. doi: 10.1038/srep22297 26923333 PMC4770439

[27] Barboro P Rubagotti A Orecchia P . Differential proteomic analysis of nuclear matrix in muscle-invasive bladder cancer: potential to improve diagnosis and prognosis. Cell Oncol Off J Int Soc For Cell Oncol. (2008) 30:13–26. doi: 10.1155/2008/686940 18219107 PMC4618805

[28] Yu T Zhao Y Hu Z Li J Chu D Zhang J . MetaLnc9 facilitates lung cancer metastasis via a PGK1-activated AKT/mTOR pathway. Cancer Res. (2017) 77:5782–94. doi: 10.1158/0008-5472.CAN-17-0671 28923857

[29] Cheng R Zhu S Guo S Min L Xing J Guo Q . Downregulation of NONO induces apoptosis, suppressing growth and invasion in esophageal squamous cell carcinoma. Oncol Rep. (2018) 39:2575–83. doi: 10.3892/or.2018.6334 29620226 PMC5983930

[30] Xia QY Wang Z Chen N Gan HL Teng XD Shi SS . Xp11.2 translocation renal cell carcinoma with NONO-TFE3 gene fusion: morphology, prognosis, and potential pitfall in detecting TFE3 gene rearrangement. Modern Pathol: Off J United States Can Acad Pathol Inc. (2017) 30:416–26. doi: 10.1038/modpathol.2016.204 27934879

[31] Raguraman R Shanmugarama S Mehta M Elle Peterson J Zhao YD Munshi A . Drug delivery approaches for HuR-targeted therapy for lung cancer. Adv Drug Delivery Rev. (2022) 180:114068. doi: 10.1016/j.addr.2021.114068 34822926 PMC8724414

[32] Shi J Wang L Zeng X Xie C Meng Z Campbell A . Precision-engineered PROTACs minimize off-tissue effects in cancer therapy. Front Mol Biosci. (2024) 11:1505255. doi: 10.3389/fmolb.2024.1505255 39649701 PMC11621628

[33] Kassabri L Benhamou RI . Druglike Molecular Degraders of the Oncogenic RNA-Binding Protein HuR. JACS Au. (2025) 5(8):3879–3891. doi: 10.1021/jacsau.5c00551 40881403 PMC12381739

[34] Jin J Wu Y Zhao Z Wu Y Zhou YD Liu S . Small-molecule PROTAC mediates targeted protein degradation to treat STAT3-dependent epithelial cancer. JCI Insight. (2022) 7(22):e160606. doi: 10.1172/jci.insight.160606 PMC9746828 36509291

