# Supplementary material for: NONO modulates tumorigenesis and metastatic potential of lung squamous cell carcinoma
Source: Front Oncol. 2026 Sep 23;16:1574103. doi: 10.3389/fonc.2026.1574103 (PMC13642221; doi:10.3389/fonc.2026.1574103)
Supplement: Supplementary file 1 [file DataSheet1.pdf]

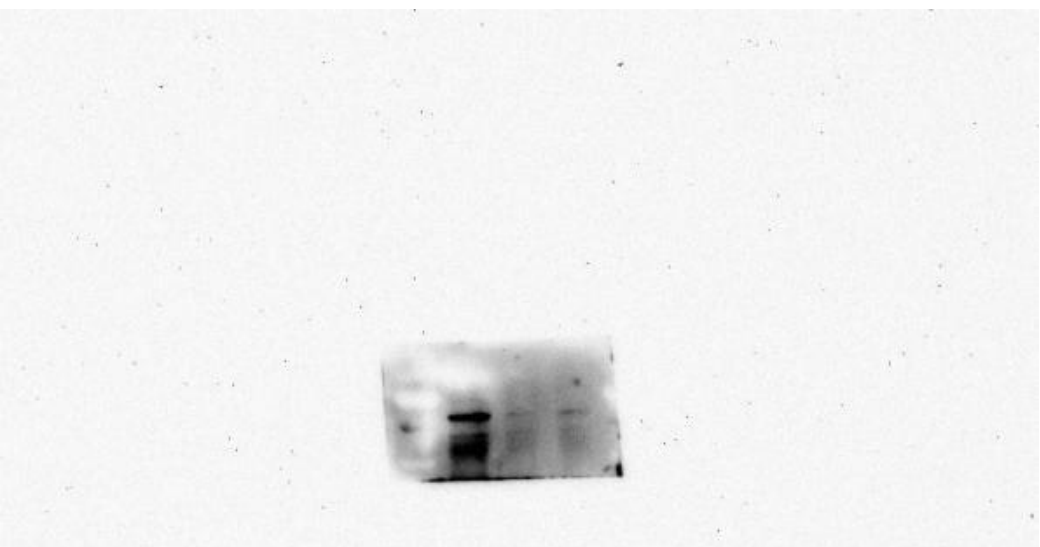

Fig2A SK-MES-1 Cell NONO

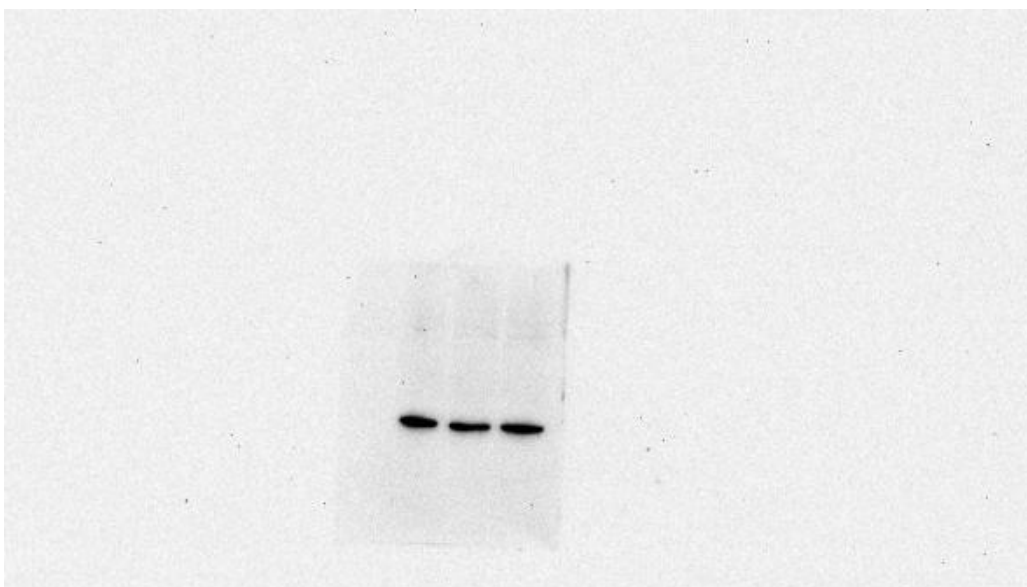

Fig2A SK-MES-1 Cell GAPDH

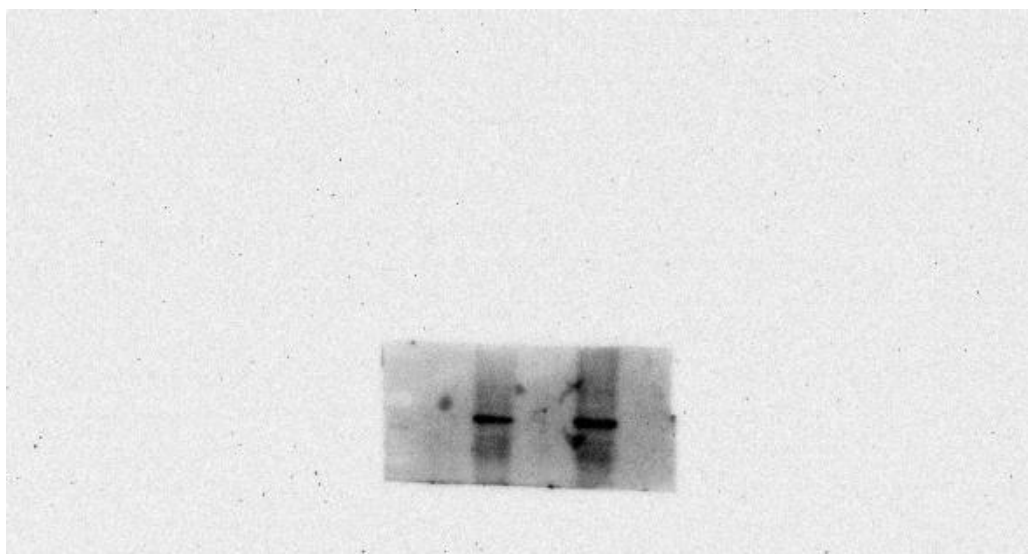

Fig4A SK-MES-1 Cell NONO

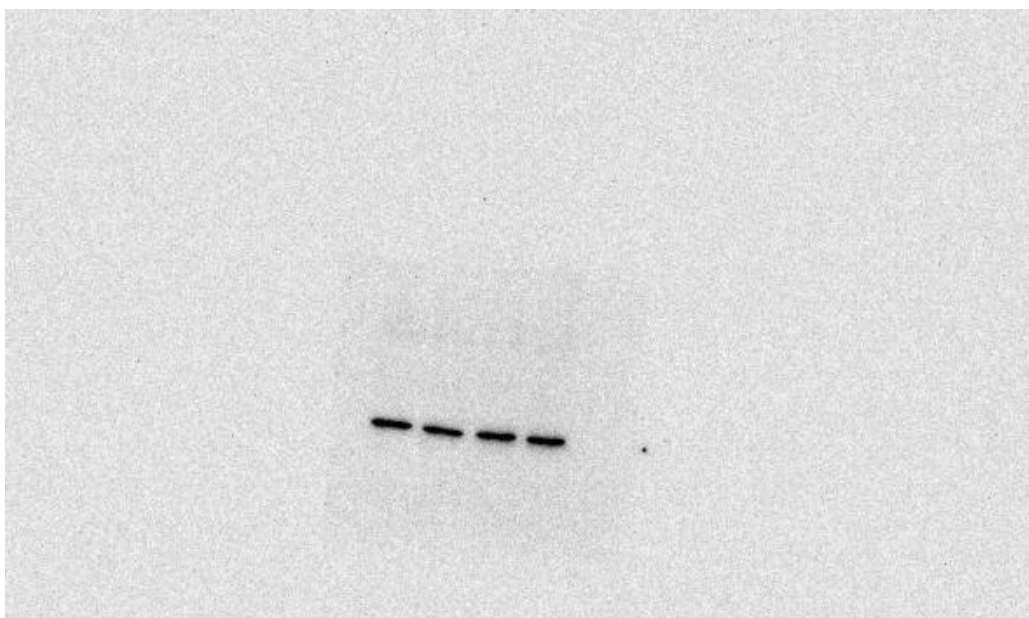

Fig4A SK-MES-1 Cell GAPDH

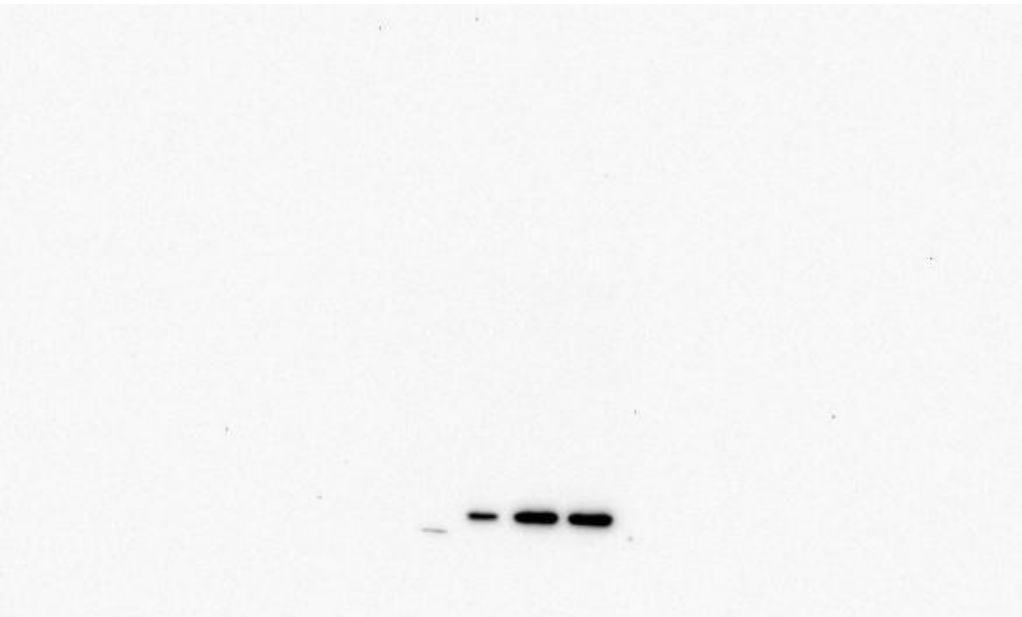

Fig6A SK-MES-1 CellBax

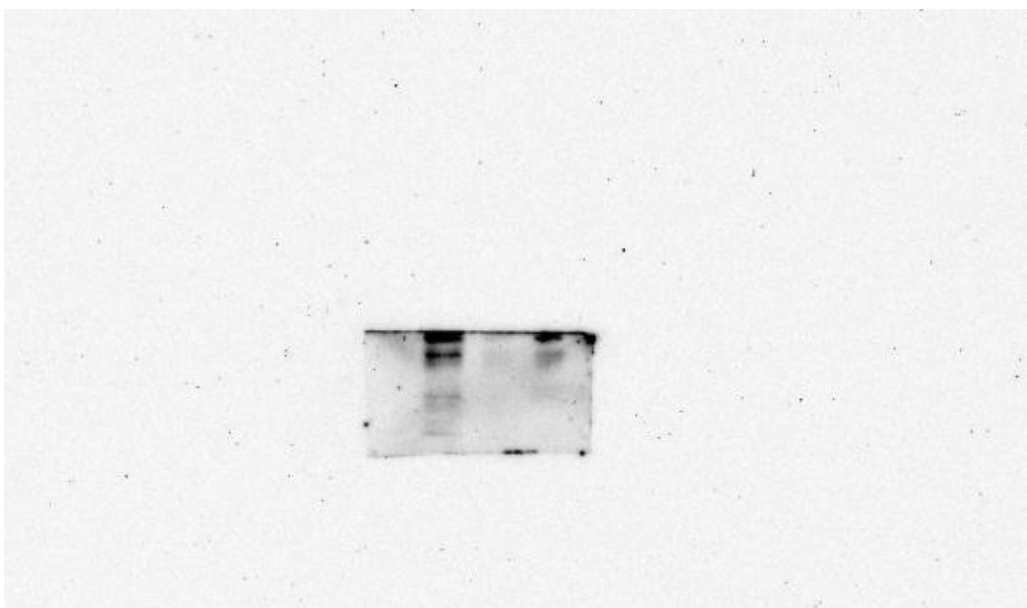

Fig6A SK-MES-1 Cell Bcl2

Fig6A SK-MES-1 Cell PARP

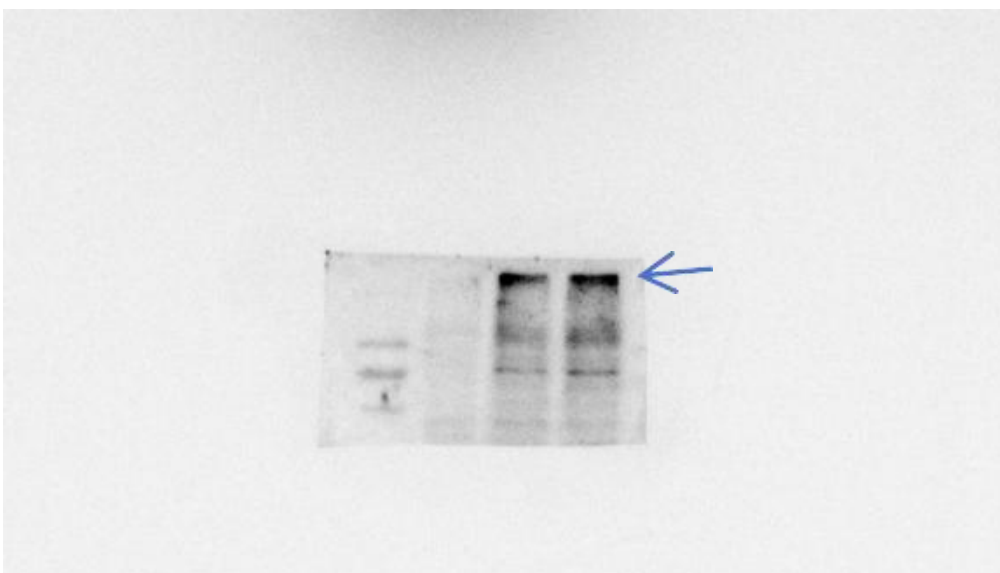

Fig6A SK-MES-1 Cell Cleaved PARP

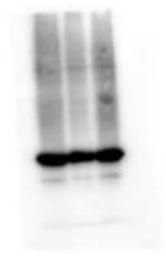

Fig6A SK-MES-1 CellGAPDH

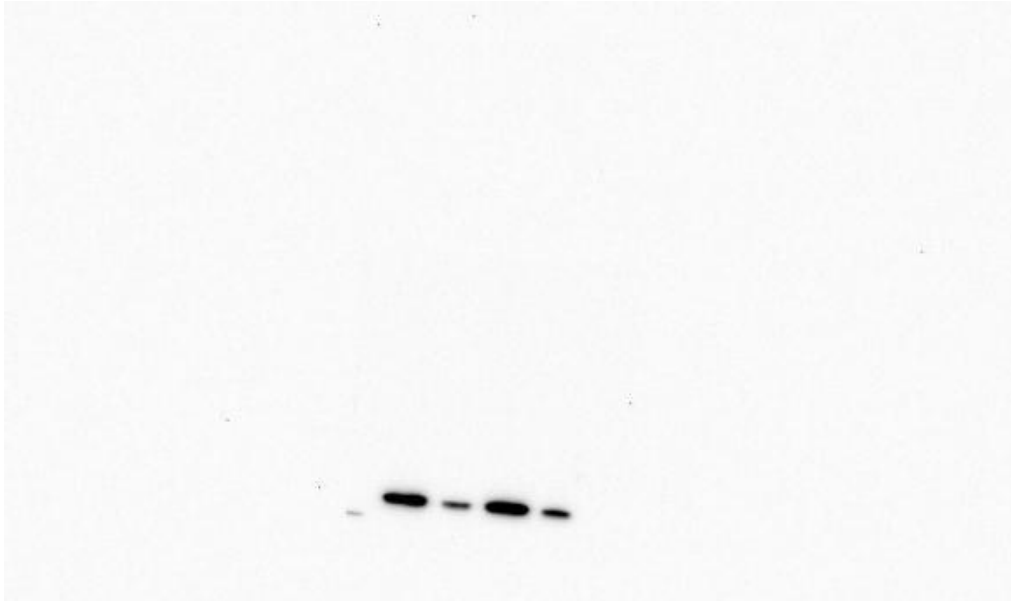

Fig6BSK-MES-1 Cell Bax

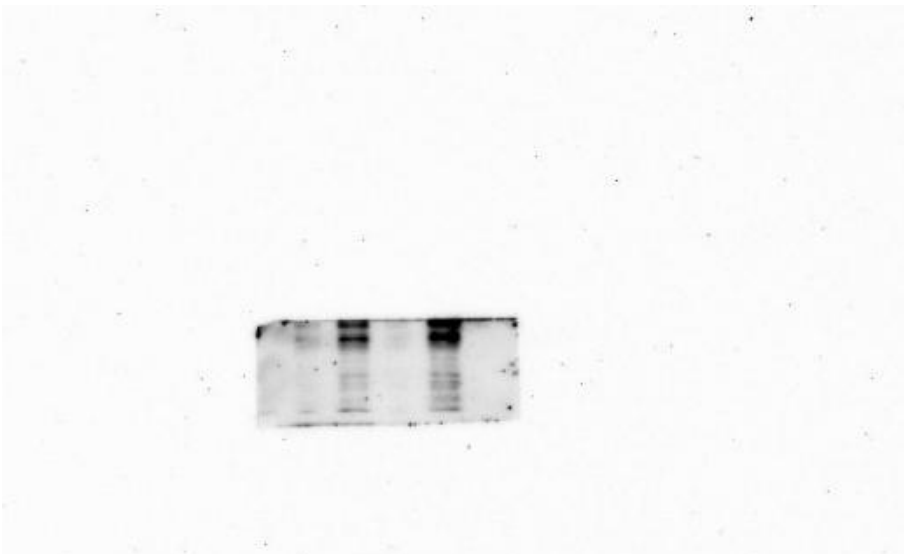

Fig6B SK-MES-1 Cell Bcl2

Fig6BSK-MES-1 Cell PARP

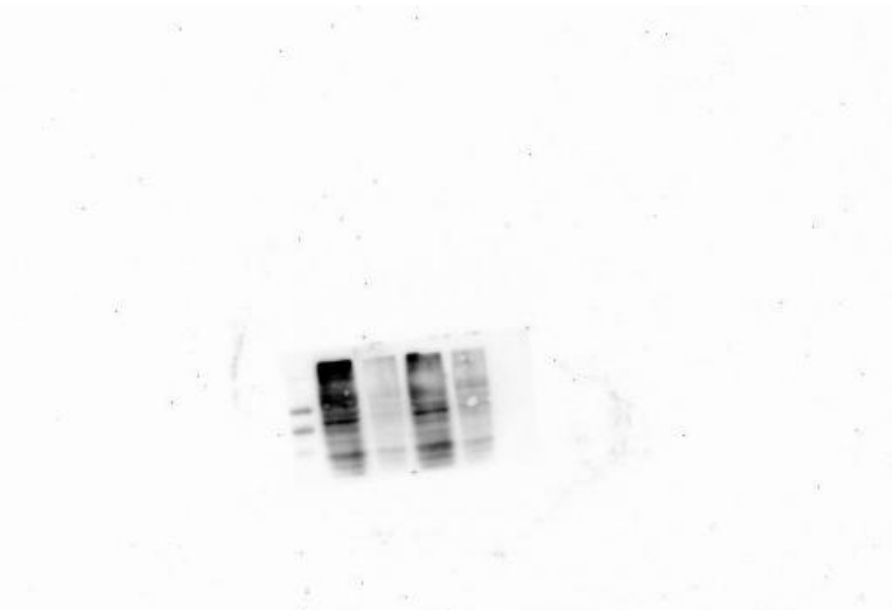

Fig6B SK-MES-1 Cell Cleaved

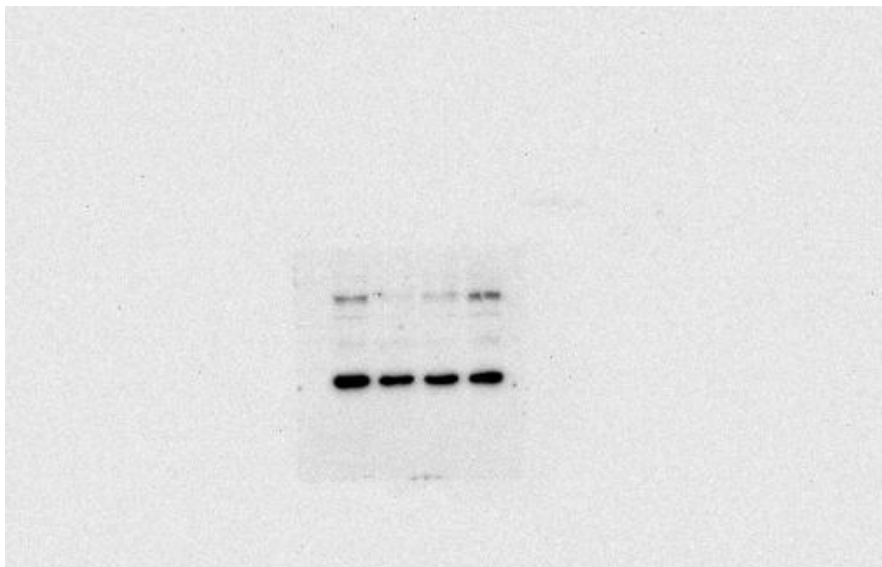

Fig6B SK-MES-1 Cell GAPDH

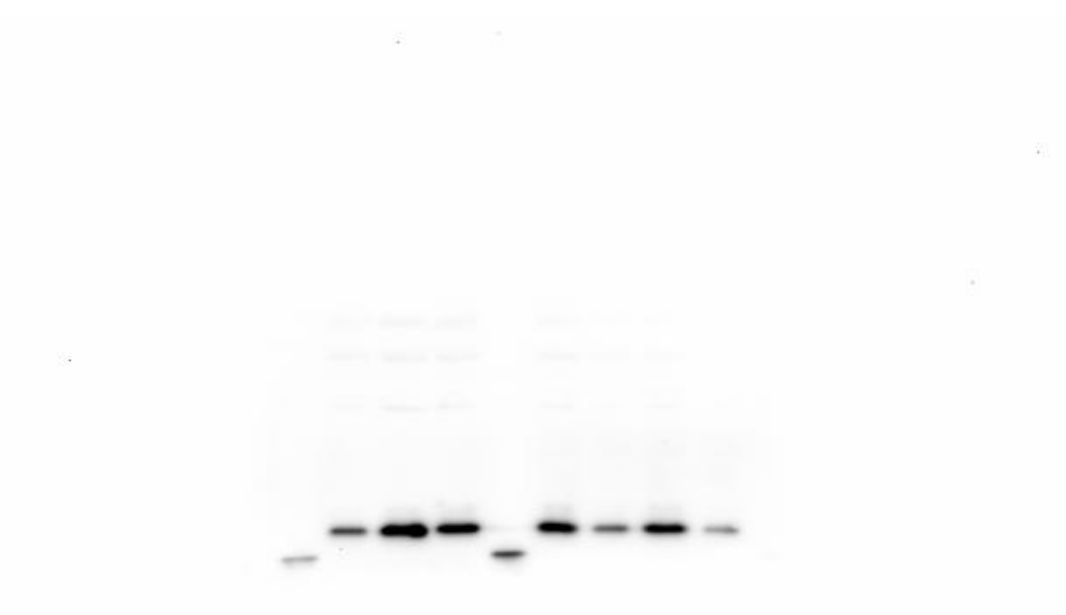

Fig6A,6C NCI-H520 cell Bax

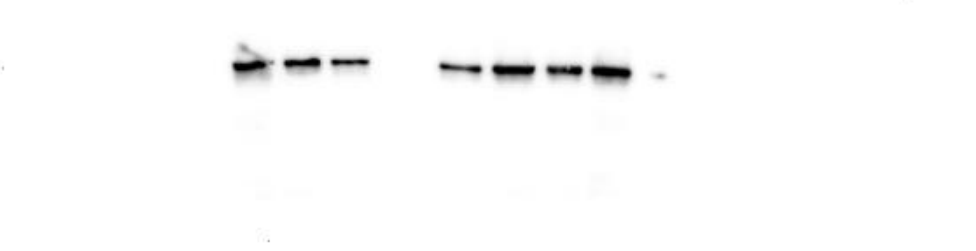

Fig6A,6C NCI-H520 cell PARP

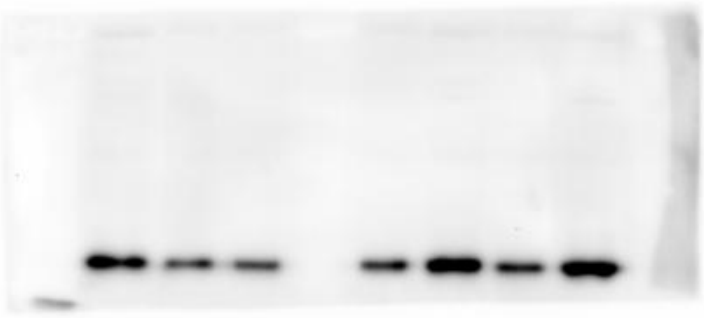

Fig6A,6C NCI-H520 cell Bcl-2

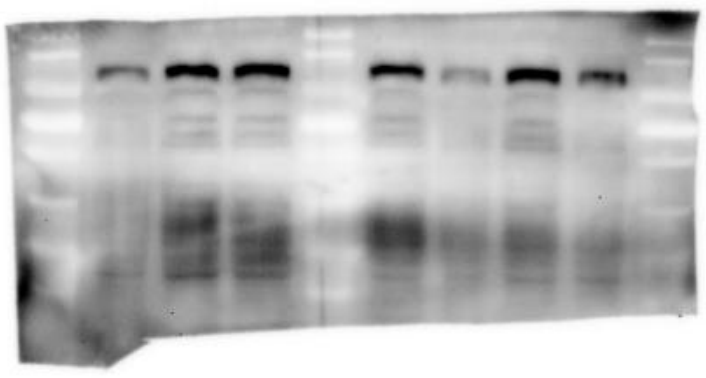

Fig6A,6C NCI-H520 cell cleaved PARP

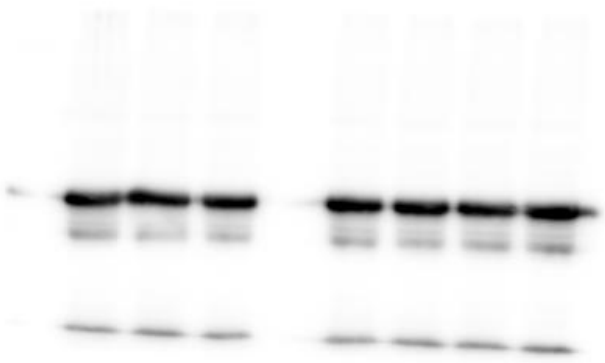

Fig6A,6C NCI-H520 GAPDH

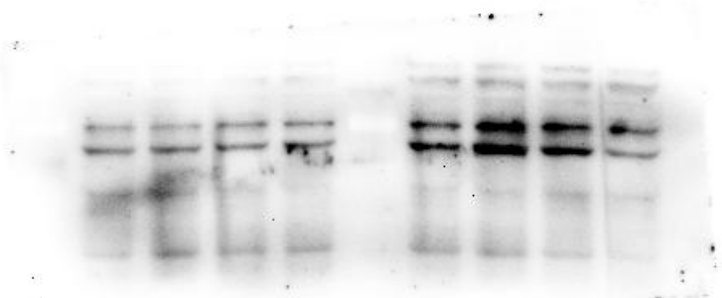

Fig6E,6G SK-MES-1 and NCI-H520 cell AKT

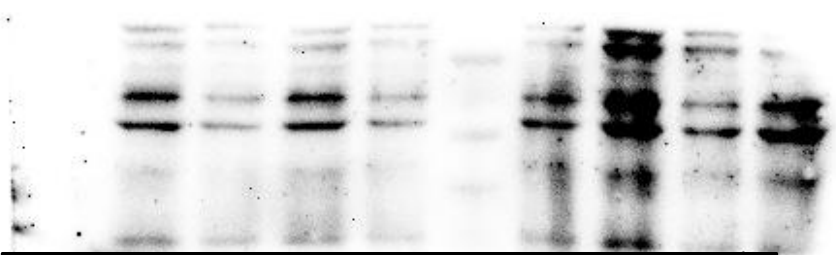

Fig6E,6G SK-MES-1 and NCI-H520 cell p-AKT

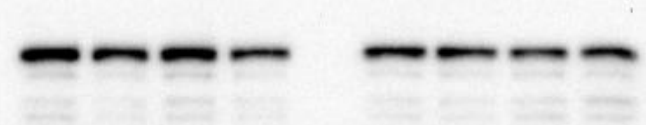

Fig6E,6G SK-MES-1 and NCI-H520 cell PI3K

Fig6E,6G SK-MES-1 and NCI-H520 cell p-PI3K

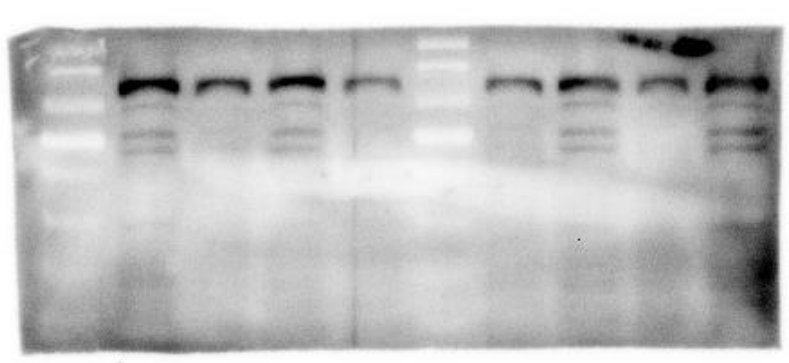

Fig6E,6G SK-MES-1 and NCI-H520 cell p-FOXO1

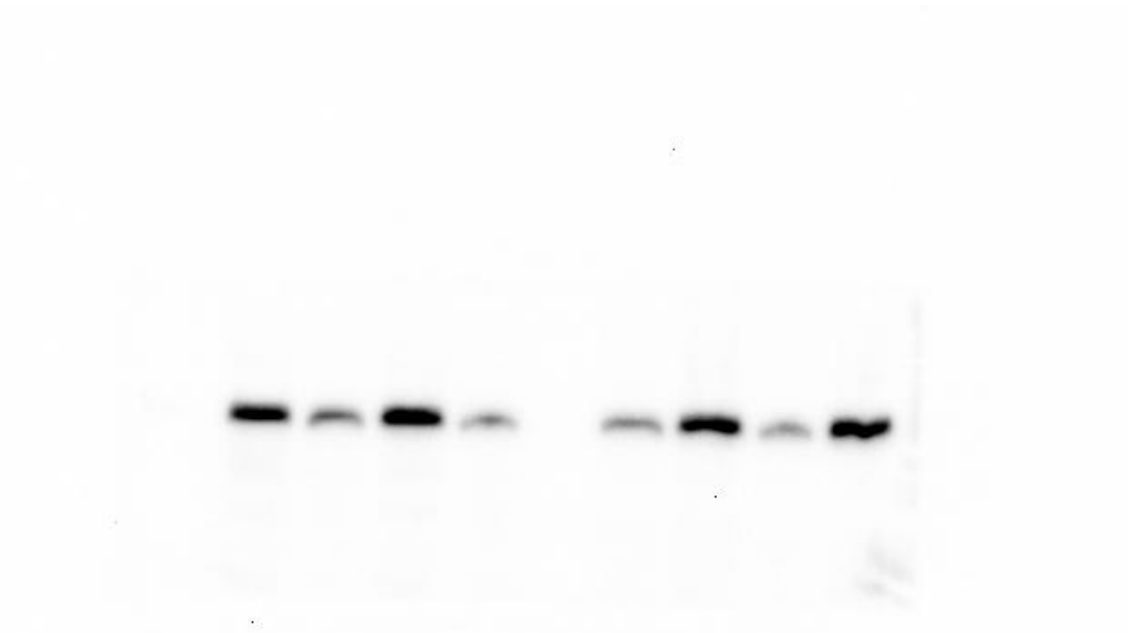

Fig6E,6G SK-MES-1 and NCI-H520 cell p-GSK3β

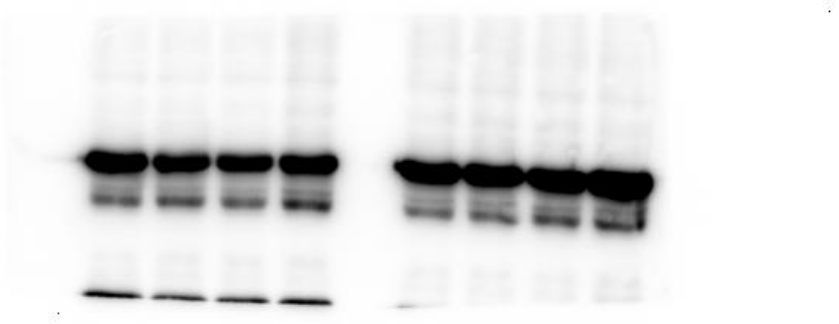

Fig6E,6G SK-MES-1 and NCI-H520 cell GAPDH

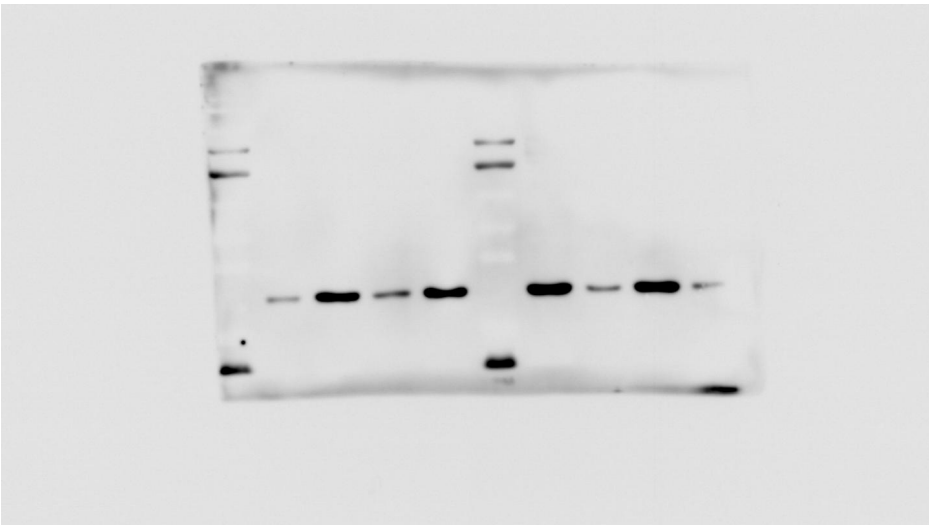

Fig6E,6G SK-MES-1 and NCI-H520 cell PUMA

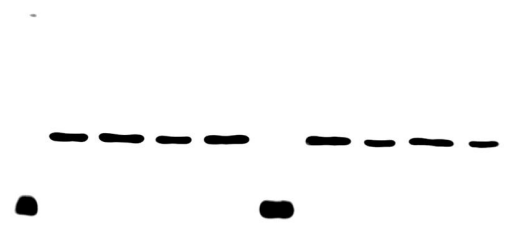

Fig6E,6G SK-MES-1 and NCI-H520 cell CDKN1A

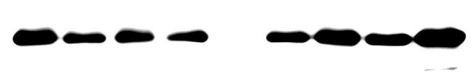

Fig6E,6G SK-MES-1 and NCI-H520 cell CDKN1B

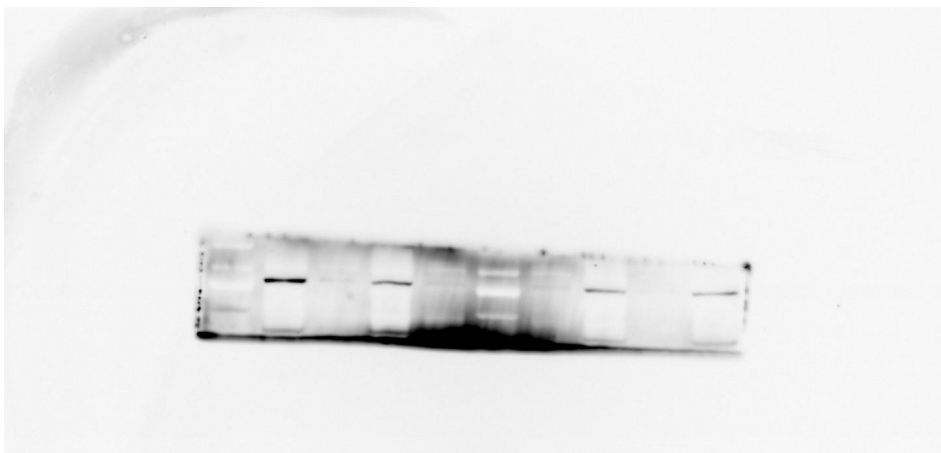

Fig6E,6G SK-MES-1 and NCI-H520 cell FOXO1

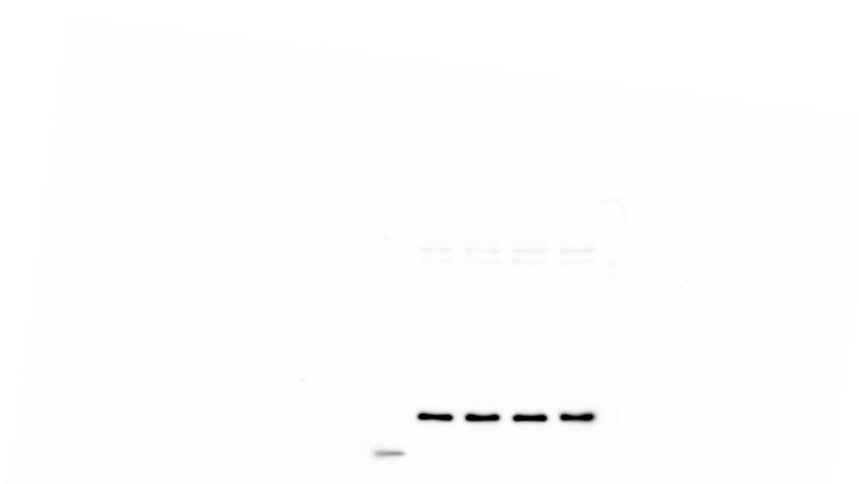

Fig6G SK-MES-1 and NCI-H520 cell Histone H3

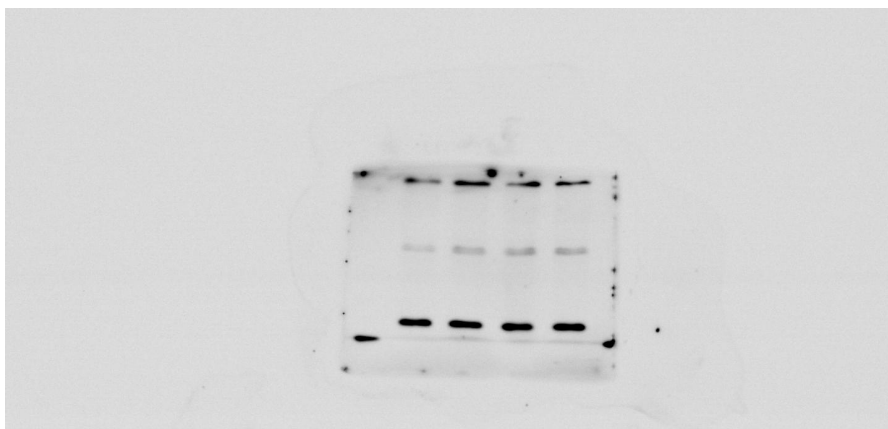

Fig6E SK-MES-1 and NCI-H520 cell Histone H3

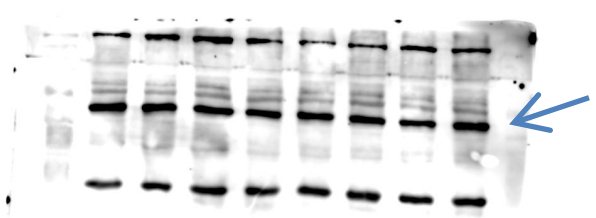

Fig7A SK-MES-1 and NCI-H520 cell AKT

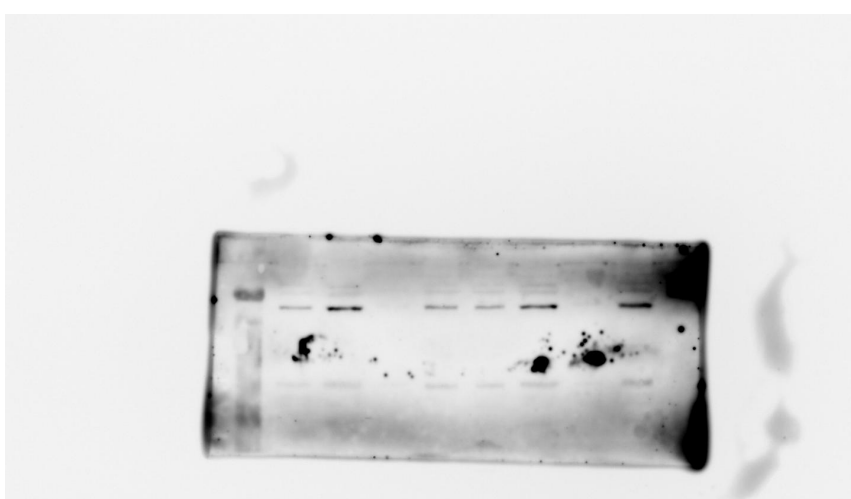

Fig7A SK-MES-1 and NCI-H520 cell p-AKT

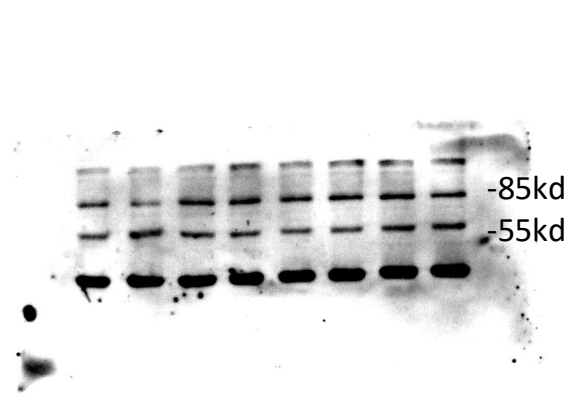

Fig7A SK-MES-1 and NCI-H520 cell PI3K

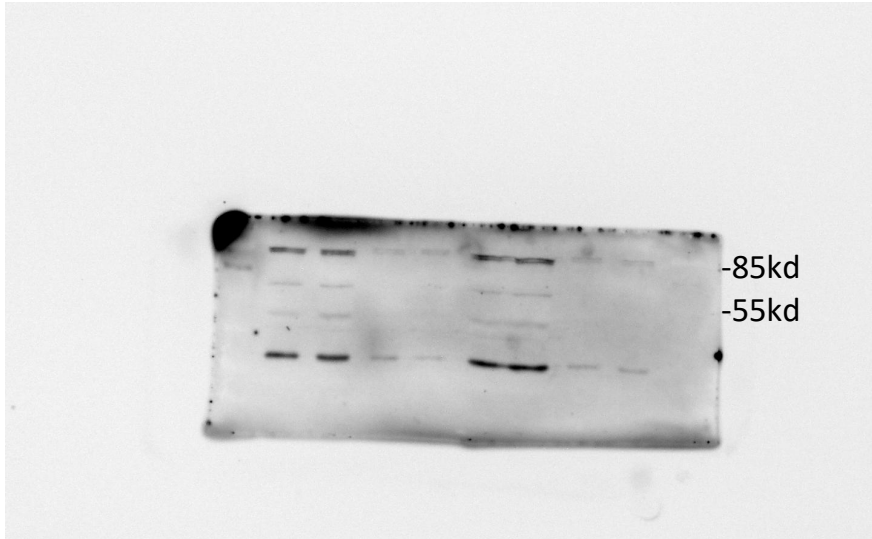

Fig7A SK-MES-1 and NCI-H520 cell p-PI3K

Fig7A SK-MES-1 and NCI-H520 cell p-GSK3 β

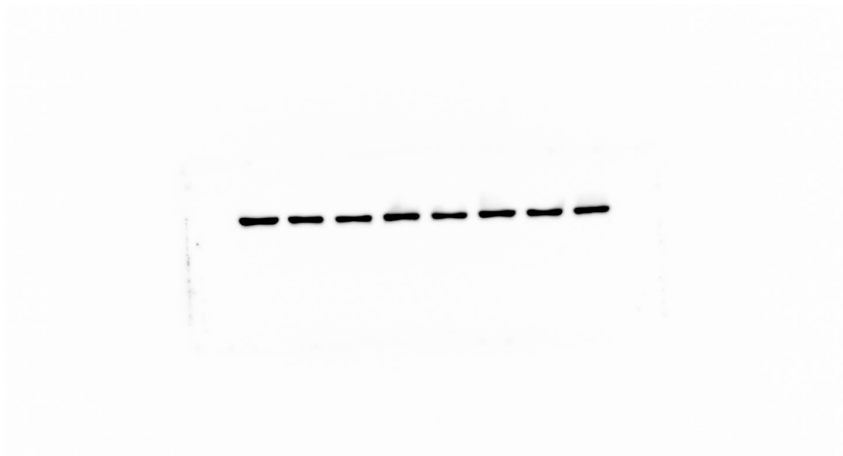

Fig7A SK-MES-1 and NCI-H520 cell GAPDH

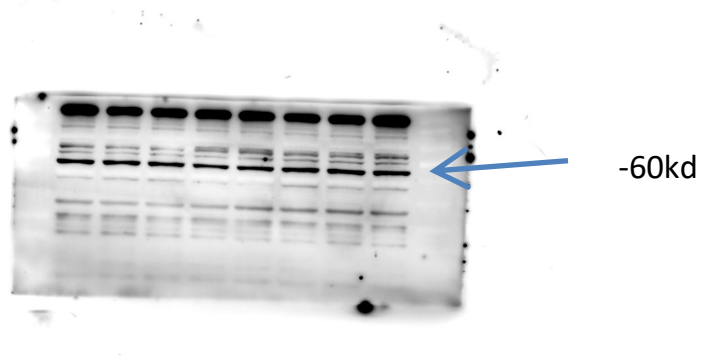

Fig7B SK-MES-1 and NCI-H520 cell AKT

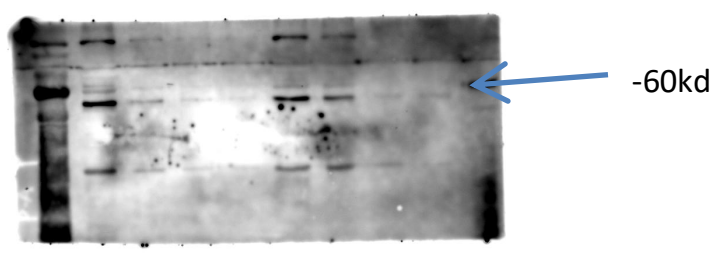

Fig7B SK-MES-1 and NCI-H520 cell p-AKT

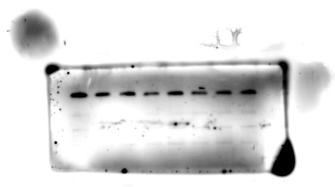

Fig7B SK-MES-1 and NCI-H520 cell PI3K

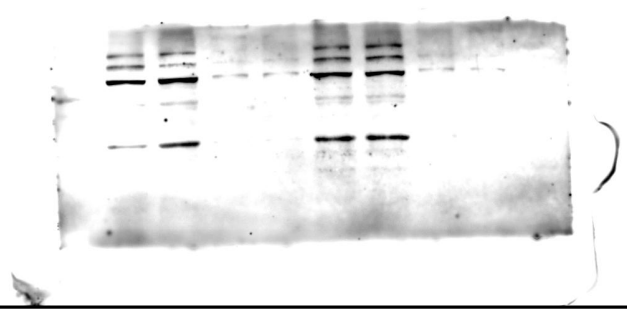

Fig7B SK-MES-1 and NCI-H520 cell p-PI3K

Fig7B SK-MES-1 and NCI-H520 cell p-GSK3 β

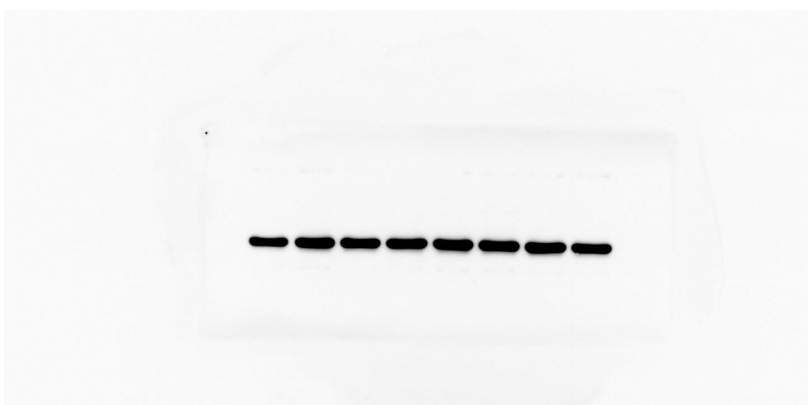

Fig7B SK-MES-1 and NCI-H520 cell GAPDH
